# Active involvement in scientific research of persons living with dementia and long-term care users: a systematic review of existing methods with a specific focus on good practices, facilitators and barriers of involvement

**DOI:** 10.1186/s12877-024-04877-7

**Published:** 2024-04-09

**Authors:** Janneke M. Groothuijse, Lisa S. van Tol, C. C. M. (Toos) Hoeksel-van Leeuwen, Johannes J. M. van Delden, Monique A. A. Caljouw, Wilco P. Achterberg

**Affiliations:** 1 Department of Public Health and Primary Care, Leiden University Medical Center, P.O. Box 9600, 2300 RC Leiden, the Netherlands; 2 University Network for the Care Sector Zuid-Holland, Leiden University Medical Center, Leiden, The Netherlands; 3 Department of Medical Humanities, University Medical Center, Utrecht, The Netherlands

**Keywords:** Involvement in research, Dementia, Long-term care, Older residents, Review, Methods

## Abstract

**Background:**

Active involvement of persons living with dementia (PLWD) and long-term care (LTC) users in research is essential but less developed compared to other patient groups. However, their involvement in research is not only important but also feasible. This study aims to provide an overview of methods, facilitators, and barriers for involving PLWD and LTC users in scientific research.

**Methods:**

A systematic literature search across 12 databases in December 2020 identified studies involving PLWD, LTC users, or their carers beyond research subjects and describing methods or models for involvement. Qualitative descriptions of involvement methods underwent a risk of bias assessment using the Critical Appraisal Skills Programme (CASP) Qualitative Checklist 2018. A data collection sheet in Microsoft Excel and thematic analysis were used to synthesize the results.

**Results:**

The eighteen included studies delineated five core involvement methods spanning all research phases: advisory groups, formal and informal research team meetings, action groups, workshops, and co-conducting interviews. Additionally, two co-research models with PLWD and carers were found, while only two studies detailed LTC user involvement methods. Four distinct involvement roles were identified: consulting and advisory roles, co-analysts, co-researchers, and partners. The review also addressed barriers, facilitators, and good practices in the preparation, execution, and translation phases of research, emphasizing the importance of diversity, bias reduction, and resource allocation. Trust-building, clear roles, ongoing training, and inclusive support were highlighted.

**Conclusions:**

Planning enough time for active involvement is important to ensure that researchers have time to build a trusting relationship and meet personal needs and preferences of PLWD, LTC users and carers. Researchers are advised not to presume the meaning of burden and to avoid a deficit perspective. A flexible or emergent design could aid involved persons’ ownership of the research process.

**Trial registration:**

Prospero 2021: CRD42021253736.

**Supplementary Information:**

The online version contains supplementary material available at 10.1186/s12877-024-04877-7.

## Background

In research characterized by active involvement, the target group plays a pivotal role in shaping research decisions and outcomes, directly impacting them. Involving patients in health research offers significant benefits [1, 2]: it enhances participant recruitment [2], refines research questions [2], aligns study results with the target population [1, 2], and promotes effective implementation of findings [1]. Active involvement of patients has also benefits for themselves, namely an enhanced understanding of research, building relationships, personal development, improved health and wellbeing, and enjoyment and satisfaction [3, 4]. It gives them a sense of purpose and satisfaction through their tangible impact.

However, for long-term care (LTC) users and persons living with dementia (PLWD) active involvement in research is less developed than for other patient groups [5, 6]. PLWD and LTC users share similar care needs, encompassing assistance with activities of daily living (ADLs), medication management, medical condition monitoring, and emotional support. Furthermore, a substantial portion of LTC users comprises individuals living with dementia [7]. Additionally, statistical data from the United States reveals that one in four older individuals is likely to reside in long-term care (LTC) facilities [8], and approximately forty to eighty percent of LTC residents in the United States, Japan, Australia, and England experience dementia or severe memory problems [7, 9].

Due to these considerations, we have chosen to combine the target audiences of PLWD and LTC users in our systematic review. However, it's important to note that while there are potential advantages to combining these target groups, there may also be challenges. PLWD and LTC users may have varying needs, preferences, and experiences, including differences in care requirements driven by individual factors like the stage of dementia, coexisting conditions, and personal preferences. Therefore, it's imperative to conduct comprehensive research and involve these communities to ensure that involvement approaches are not only inclusive but also tailored to meet their specific requirements.

Given our ageing population and the intricate health challenges faced by PLWD and LTC users, including their vulnerability and shorter life expectancy in old age, it's crucial to establish effective research involvement methods. These individuals have unique needs and preferences that require attention. They possess a voice, and as researchers, it is our responsibility to not only listen to them but also actively involve them in the research process. Consequently, it is essential to identify means through which the voices of PLWD and LTC users can be effectively heard and ensure that their input is incorporated into research.

Fortunately, publication of studies on involvement of PLWD and LTC users in scientific research is slowly increasing [5, 9–11]. A few reviews have described how PLWD and LTC users were involved [5, 9, 10]. However, with the increasing attention for involvement, the understanding of when involvement is meaningful grows and stricter requirements can be imposed to increase the quality of active involvement [12, 13]. To our knowledge there is no up to date overview of involvement methods used with either or both PLWD and LTC users. Such an overview of involvement methods for PWLD and LTC users would provide a valuable, comprehensive resource encompassing various stages of the research cycle and different aspects of involvement. It would equip researchers with the necessary guidance to navigate the complexities of involving PLWD and LTC users in their research projects.

Recognizing the need to enhance the involvement of PLWD and LTC users in scientific research, this systematic review aims to construct a comprehensive overview of the multiple methodologies employed in previous studies, along with an examination of the facilitators and barriers of involvement. Our overarching goal is to promote inclusive and effective involvement practices within the research community. To achieve this objective, this review will address the following questions: (1) What kind of methods are used and how are these methods implemented to facilitate involvement of PLWD and LTC users in scientific research? (2) What are the facilitators and barriers encountered in previous research projects involving PLWD and LTC users?

## Methods

### Protocol and registration

The search and analysis methods were specified in advance in a protocol. The protocol is registered and published in the PROSPERO database with registration number CRD42021253736. The search and analysis methods are also described below more briefly.

### Information sources, search strategy, and eligibility criteria

In preparation of the systematic literature search, key articles and reviews about involvement of PLWD and LTC users in research were screened to identify search terms. In addition, Thesaurus and MeSH terms were used to broaden the search. The search was conducted on December 10, 2020, across multiple databases: PubMed, Medline, Embase, Emcare, Web of Science, Cochrane Library, PsycINFO, Academic Search Premier, JSTOR, Social Services Abstracts, Sociological Abstracts, Psychology and Behavioral Sciences Collection. The search terms were entered in "phrases". The search strategy included synonymous and related terms for dementia, LTC user, involvement, research, method, and long-term care. The full search strategy is provided in supplement 1.

After conducting the search, records underwent initial screening based on titles and abstracts. Selected reports were retrieved for full-text assessment, and studies were evaluated for eligibility based on several criteria. However, no restriction was made regarding publication date. First, to be included studies had to be written in English, German, French, or Dutch. Second, we only included original research studies. Third, studies were excluded when the target group or their representatives were not involved in research, but only participated as research subjects. Fourth, studies were excluded when not describing involvement in research. Therefore, studies concerning involvement in care, policy, or self-help groups were excluded. Fifth, the focus of this systematic review is on methods. Therefore, studies with a main focus on the results, evaluation, ethical issues, and impact of involvement in research were excluded. Additionally, we have not set specific inclusion or exclusion criteria based on study design since our primary focus is on involvement methodologies, regardless of the chosen research design. Sixth, the included studies had to concern the involvement in research of PLWD or adult LTC users, whether living in the community or in institutional settings, as well as informal caregivers or other representatives of these groups who may represent PLWD and LTC users facing limitations. Studies that involved LTC users that were children or ‘young adults’, or their representatives, were excluded. Studies were also excluded if they involved mental healthcare users if it remained unclear if the care that they received entailed more than only treatment from mental healthcare providers, but for example also assistance with ADL.

### Terminology

For readability purposes, we use the abbreviation PLWD to refer to persons diagnosed with dementia, and we use the abbreviation LTC users to refer to persons receiving long-term care, at home or as residents living in nursing homes or other residential facilities. We use the term carers to refer to informal caregivers and other representatives of either PLWD or LTC users. As clear and consistent definitions regarding participatory research remains elusive [14, 15], we formulated a broad working definition of involvement in research so as not to exclude any approach to participatory research. We defined involvement in research as “research carried out ‘with’ or ‘by’ the target group” [16], where the target group or their representatives take part in the governance or conduct of research and have some degree of ownership of the research [12]. It concerns involvement in research in which lived experienced experts work alongside research teams. We use the terms participation and participants, to refer to people being part of the research as study subjects.

### Selection process, data-collection process, and data items

Titles and abstracts were independently screened by the first and second author (JG and LT). Only the studies that both reviewers agreed and met the inclusion criteria were included in the full-text screening process. Any uncertainty about whether the studies truly described a model or approach for involvement, was resolved by a quick screening of the full-text paper. The full-text screening process was then conducted according to the same procedure by JG and LT. Any disagreement was resolved by discussion until consensus was reached. If no agreement could be reached, a third researcher (MC) was consulted. References of the included studies were screened for any missing papers.

The following information was collected on a data collection sheet in Microsoft Excel: year and country of publication, topic, research aim, study design, living situation of involved persons (at home or institutionalized), description of involved persons, study participants (study subjects), theories and methods used, type/role(s) of involvement, research phase(s), recruitment, consent approach, study setting, structure of participatory activities, training, resources, facilitators, barriers, ethics, benefits, impact, and definition of involvement used.

JG independently extracted data from all included studies, the involved co-researcher (THL) independently extracted data from two studies, the second author (LT) from five. Differences in the analysis were discussed with the co-researcher (THL) and second author (LT) until consensus was reached. As only minor differences emerged, limited to the facilitator and barrier categories, data from the remaining studies was extracted by JG.

### Risk of bias assessment

Every research article identified through the systematic review exclusively comprised qualitative descriptions of the involvement method(s) employed. Consequently, all articles underwent evaluation using the Critical Appraisal Skills Programme (CASP) Qualitative Checklist 2018 [17], as opposed to the checklists intended for quantitative or mixed methods research. All included studies were independently assessed on quality by two reviewers (JG,LT) and any disagreement was resolved by discussion until consensus was reached. The CASP Qualitative Checklist consists of ten questions. The checklist does not provide suggestions on scoring, the first author designed a scoring system: zero points if no description was provided (‘no’), one point if a minimal description was provided (‘can’t tell’) and two points when the question was answered sufficiently (‘yes’). The second question of the checklist, “is a qualitative methodology appropriate”, was not applicable to the aims (i.e., to describe involvement) of the included studies and was therefore excluded. The tenth question was translated into a ‘yes’, ‘can’t tell’, or ‘no’ score to fit the scoring system. A maximum of eighteen points could be assigned.

### Synthesis methods

Tables were used to summarize the findings and to acquire an overview of (1) the kinds of methods used to enable involvement of PLWD, LTC users, or carers in scientific research, and (2) the facilitators and barriers for involving this target group in scientific research. As to the first research aim, the headings of the first two tables are based on the Guidance for Reporting Involvement of Patients and the Public, long form version 2 (GRIPP2-LF) [18]. Because our systematic review focusses on methods, only the topics belonging to sections two, three, and four were included. Following Shippee et al., three main research phases were distinguished: preparation, execution, and translation [19]. Furthermore, the following fields were added to the GRIPP2-LF: First author, year of publication, country of study, setting of involvement, frequency of meetings, and a summary description of activities.

Concerning the second research aim, the extracted facilitators, barriers, and good practices were imported per study in ATLAS.ti for qualitative data analysis. Following the method for thematic synthesis of qualitative studies in systematic reviews [20], all imported barriers, facilitators and good practices were inductively coded staying 'close' to the results of the original studies, which resulted in 50 initial codes. After multiple rounds of pile sorting [21], based on similarities and differences and discussions in the research team, this long code list was grouped into a total of 27 categories, which were thereafter subsequently organized into 14 descriptive themes within the three research phases (preparation, execution, translation).

## Results

### Study selection and characteristics

**Fig. 1:**
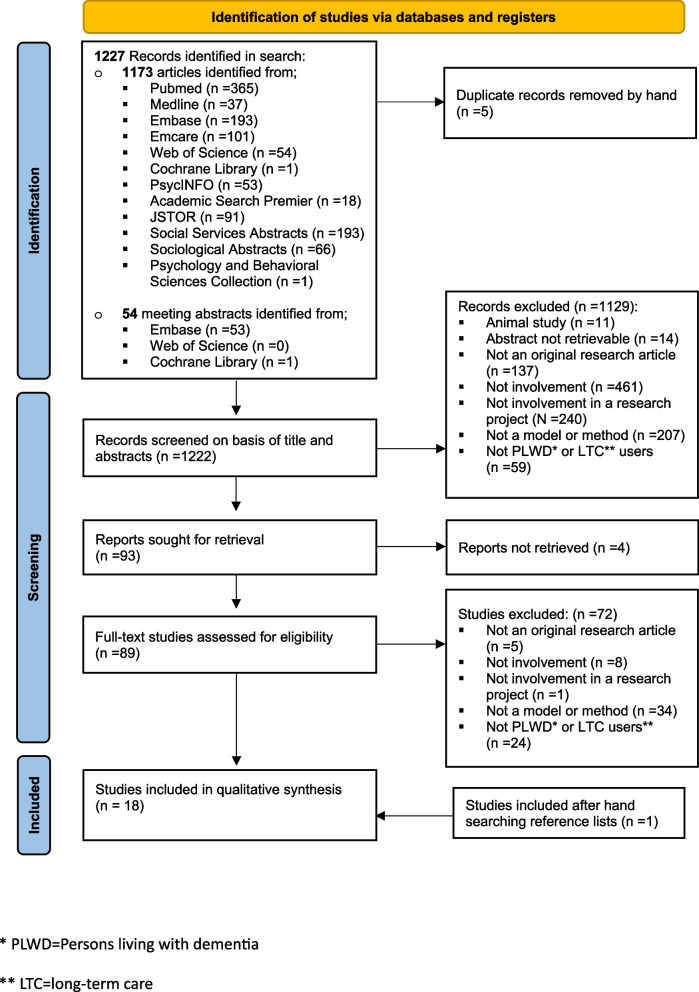
Preferred Reporting items for Systematic Reviews and Meta-analyses (PRISMA) flow diagram

The Prisma Flow Diagram was used to summarize the study selection process [22]. In the full text screening, 72 of the 93 remaining studies were excluded because they were not original research articles (n = 5), not about involvement (n = 8), not about involvement in a research project (n = 1), they did not describe a model or method for involvement (n = 34), or they were not about PLWD or LTC users (n = 24). The search resulted in 18 publications eligible for analysis (Fig. 1).

Table 1 presents the general study characteristics. Two studies explicitly aimed to develop a model for involvement or good practice, and both focus on co-research either with PLWD [23] or their carers [13]. The other sixteen provide a description of the involvement of PLWD [24–34] or LTC users in their research projects [35–39].

**Table 1:** General characteristics of included studies

First Author, year, country of study	Topic of study	People involved (lived experienced experts)	Setting of involvement	Study subjects	Theoretical underpinnings of involvement	Methods used	Quality score
Baur and Abma, 2012, NL [35]	Collective involvement through relational empowerment to improve the mealtime experience in a residential home	7 older female residents all aged over 80, all with a physical disability or illness. 4 involved persons lived in residential care apartments, the other 3 lived in sheltered accommodation	Residential home	n/a	Participatory action research and appreciative inquiry	Homogenous action group, heterogenous dialogue meetings, larger resident meeting	17/18
Beukema and Valkenberg, 2007, NL [36]	Implementation of a demand-driven approach to elderly care through exemplarian action research in five nursing homes	? representatives of clients ? managers ? workers	In five homes for elderly care	n/a	Exemplarian action research	Steering committees, training sessions, interviews, participatory observation, group discussions, working groups	12/18
Brown et al. 2017, UK [37]	Critical reflection on involvement of the public in research on intimacy and sexuality in care homes, and development of recommendations for involvement in research	2 older community representatives, one male and one female. One was aged 81 years, the other 74 years	Skype, public places such as cafes, and at the university	? interviewees ? focus group participants	Public involvement and service-user involvement	Research team meetings, email discussions, evaluative interviews, workshop	15/18
Clarke et al. 2018, UK [24]	Secondary data analysis of qualitative data in partnership with persons living with dementia	34 people met in four groups of 1–8 people, with experience of living with dementia, either personally (n≈21) or as a family caregiver (n≈10). All involved persons lived in their own home or had moved to live with a relative	Groups met in their normal peer support group location	106 people living with dementia	Participatory research and responsive research	Participatory secondary data analysis workshops	13/18
Di Lorito et al. 2020, UK [13]	To propose a model for good practice in co-researching with carers of people living with dementia	2 people with lived experience of caring for someone living with dementia	At the university, homes of people living with dementia and informal settings	14 people living with dementia and their carers	Patient and Public involvement	Meetings, training sessions, co-research interviews, data-analysis, personal diaries, workshop Research Cycle Model for good practice in co-research	18/18
Flavin and Sinclair, 2019, AU [25]	Consumer involvement in a research project investigating supported decision making among people living with dementia	Three advisory groups with in total: 3 people living with dementia 3 current or former care-partners of a person living with dementia 18—45 industry and advocacy representatives	The three advisory group meetings were run in three states	n/a	Patient and Public involvement	Advisory group meetings based on consensus approach, consultation meeting	7/18
Froggatt et al. 2015, UK [38]	Integration of involvement in research into the design and delivery of a multi-site research study based in care homes	6 public involvement in research (PIR) members were involved, who all had prior personal or work experience in care homes. Reports on the activities of 5 PIR members	Six care homes	84 residents 53 care home staff 57 primary care staff 3 relatives 12 undefined stakeholder interviews + focus groups with 8 care home and primary care staff	Public involvement in research	Project management meetings, fieldwork meetings and training, assistance in interviews and focus groups, project team meetings, validation event	15/18
Giebel et al., 2019, UK [26]	Involvement of people living with dementia and their carers in a programme on effective home support in dementia	Small reference group: 8–12 informal carers (current and previous) ? people living with dementia ? lay public involvement 11–15 members of the research programme Virtual lay advisory group: 20 informal carers	Small reference group meet face-to-face (setting not described) Virtual lay advisory group: consulted through email	n/a	Public involvement in research, user/researcher roles action research	Small reference group and virtual lay advisory group	14/18
Goeman et al., 2019, AU [27]	Description of the co-design process in a project that evaluated how the key worker role can best support people living with dementia in the community setting	Expert working group: 1 person living with dementia 1 care-partner 6 representatives of partner organisations Reference group: 2 people living with dementia 1 care-partner 2 consumer representatives 7 representatives of departments/organisations/ health professionals	Teleconferences and two face-to-face interactive workshops (setting not described)	? consumers ? support workers ? organisations providing support worker models	Consumer and community involvement	Expert working group, reference group, teleconferences, face-to-face meetings, workshops	9/18
Gregory et al., 2020, EU [28]	Description of the approach to setting- up involved persons-panels in a pan-European cohort study concerning risks for developing Alzheimer's disease	34 persons who were cognitively healthy or had mild cognitive impairment were involved in 5 country-wide panels (7–12 people) (divided into local panels) and 1 central panel (6–10 people)	Local centres	2000 participants in longitudinal cohort study	Patient and Public involvement	Local and central Patient and Public involvement panels	16/18
Hanson et al., 2007, SE [29]	Development of a user-friendly technology-based support service in partnership with older people with early-stage dementia and their family members	Development group: 7 persons with early-stage dementia 1 spouse Testing group, met in five groups: 19 persons with early-stage dementia 12 family members	Local day centre	n/a	Scandinavian participatory design	Discussion groups, reviewing platform session, interviews, focus groups	16/18
Hassan et al., 2017, UK [30]	Involvement of members of the public in informing design and procurement decisions regarding connected health wearables in dementia research	Group 1: 5 people living with dementia (age > 65), 4 informal carers Group 2: 8–12 people with young onset dementia (age < 65) Group 3: 2 people with mild cognitive impairment, 1 spouse Group 4: 9 people without memory problems (age > 50)	Dementia resource centre (people living with dementia and carers), drop-in support group (Dementia < 65 yrs.), University of Manchester (no known memory problems > 50 yrs.)	n/a	Patient and Public involvement	Interactive workshops, drop-in sessions and meetings	14/18
Mann and Hung, 2019, CA [31]	Discussion of shared experiences on conducting action research together in a study concerning improvement of dementia care	A man diagnosed with Alzheimer's disease at the age of 58, 8 years prior to the research project	Medical unit (31 beds) for older people living with dementia or cognitive impairments in hospital and meetings in a coffee shop	7 patients living with dementia, 50 staff (including 30 nurses, 5 health practitioners, 15 physicians, and 1 senior administrator in leadership)	Action research, appreciative inquiry and COINED-model [23]	(only the methods used with Jim) Research meetings in coffee shop, email, education workshop	15/18
Poland et al. 2019, UK [32]	Critical evaluation of implementation of Patient and Public Involvement in promoting independence in dementia study	5 carers (co-applicant, advisory group, co-researcher in data collection) 8 people with mild to moderate dementia (co-research in data analysis, met in 2 groups of 4)	n/a	n/a	Patient and Public involvement	Co-applicant, PPI advisory group, co-research interviews, data-analysis workshops	16/18
Shura et al. 2010, USA [39]	Advance the process of culture change within long-term care and assisted living settings by engaging residents directly in the process as experts	28 female nursing home residents 9 male nursing home residents 9 female assisted living residents 3 male assisted living residents All with varying levels of physical and cognitive challenges	2 assisted living care units and 5 care units in a nursing home	n/a	Participatory action research	Seven unit specific resident/participatory action groups	12/18
Stevenson and Taylor, 2019, UK [33]	Involvement of persons living with dementia as co-researchers in qualitative analysis of risk communication in dementia care (concepts and communication)	4 persons with early-stage dementia, two male and two female; two are aged under 65, one between 70–74 years, and one between 75–79 years	Location of Alzheimer's Society Service User Review Panel	? interviews with persons living with dementia, healthcare professionals and family carers	Patient and Public involvement (INVOLVE)	Data analysis session	15/18
Swarbrick and Doors 2018, UK [23]	Development of the CO-researcher INvolvement and Engagement in Dementia (COINED) model in co-production with people living with dementia	3 inquiry groups with 4–18 members based on prior established research groups of people living with dementia	Familiar venue of pre-existing research groups	n/a	Co-operative inquiry	3 inquiry groups COINED-model	13/18
Tanner, 2012, UK [34]	Involvement of people living with dementia as co-researchers in research on experiences of transitions between care services	2 male (71 and 77) 1 female (60) All had a diagnosis of dementia and had undergone a recent transition within or between care-services. They all lived with their spouse and had mild or moderate dementia	Training in familiar location and interviews in the homes of persons living with dementia	5 interviews with people living with dementia (most were recently diagnosed)	Co-research	Training sessions, co-research interviews, team meetings	14/18

### Quality assessment

Table 1 presents the CASP-score per study [17]. Five scored 16 to 18 points [13, 28, 29, 32, 35], indicating high quality with robust methods, clear aims, and strong data analysis. Eleven scored 12 to 15 [23, 24, 26, 30, 32–34, 36–39], showing generally strong methodologies but with some limitations. Two scored 9 or lower [25, 27], signifying significant methodological and analytical shortcomings. Notably, these low-scoring studies were short articles lacking clear recommendations for involvement in research.

### Design and implementation of involvement

#### Phases and methods of involvement

Table 2 describes the involvement methods used for and the implementation of involvement in research. The included studies jointly presented methods for involvement in the three main research phases [19]. Regarding the preparation phase, which involves the preparatory work for the study, only three studies provided detailed descriptions of the methods employed [26, 30, 32]. The execution phase, encompassing the actual conduct of the research, was most frequently discussed [23–29]. Five studies addressed the translation phase [13, 25, 31, 36, 37], where the focus shifts to translating research findings into actionable outcomes.

**Table 2:** Overview of the involvement methods and their implementation of included studies

Author	Aim of involvement	Role(s) of involved persons	Phase(s) of research mainly reported on	Form of involvement	Frequency of meetings with involved persons	Description of activities
Baur and Abma, 2012 [35]	To develop a shared vision and enhance empowerment among residents to improve life in the residential home	Resident involvement as partnership	Execution^a^	Governance and conduct	Over a 7-month period, 8 homogeneous meetings were held	Involvement method: homogenous action group Five phases of non-linear progression (i.e., not straight progression from little influence to feeling empowered) 1. Phase 1: talking about experiences of living in the residential home and setting the agenda for the research project and improvements in the home. Picture cards were used to start the conversation 2. Phase 2: getting to know each other and exploring shared experiences about the meals 3. Phase 3: feeling of empowerment through discovery of shared discontent about the mealtime experience 4. Phase 4: repeated sharing of negative experiences resulted in stagnation. Following appreciative inquiry, the group made a paste-up of ideal meals which changed the group dynamic 5. Phase 5: turning discontent into constructive advice and partnership with service providers for improving meals • The action group was supported and facilitated by the first author • A meeting was arranged in which 60 other residents were involved to discuss the ideas for improvements • Four heterogenous dialogue meetings with managers and kitchen staff were organized. Through this dialogue the group developed a partnership, based on the common aim to improve the well-being of residents, with those responsible for making changes in the organisation
Beukema and Valkenberg, 2007 [36]	To develop a shared vision in order to implement contextualized demand-driven care	Co-researchers	Translation	Governance and conduct	n/a	Involvement methods: discussion sessions and working groups In each of the five locations initial contact was with management, who investigated the situation and were responsible for the implementation process. Steering committees that were responsible for the research process were set up at every location. Three stages of exemplary action research were followed: the thematic stage, crystallization stage, and the exemplar stage 1. Preparation of thematic stage: a training was organized in order to challenge the superiority of dominant view of scientific knowledge and to discuss location-specific and shared questions 2. Stage 1 Thematic stage, getting to know the situation under study: Inventory of themes that represent a shared understanding and formulating a shared vision, through interviews in an open interview setting, participatory observations, and discussion sessions. A report was prepared with a thematic representation and the results were discussed in sessions 3. Stage 2 Crystallization stage: researcher and co-researchers define the most important theme, the exemplar. Individual coaching and group discussions are important to increase reflexivity. When consensus is reached, the co-researchers draw up a plan in working groups to deal with the exemplar 4. Stage 3 Exemplar stage, implementation of developed plan: processes of intervention and evaluation are central to this stage. Methods are participatory observation, intervision, and discussion. In this stage researchers prepare to withdraw and teach co-researchers to deal with their situation themselves • Researchers held multiple roles such as discussant, trainer, coach, and consultant
Brown et al., 2017 [37]	To provide feedback on recruitment plans, themes arising from data analysis, study plans and involvement in dissemination	Community representatives	Execution and translation	Governance (and conduct in workshop)	Community representatives were invited to all research meetings	Involvement method: research team meetings and workshop The two community representatives were invited to be involved in all aspects of the project for which specific training was not considered necessary 1. Research group meetings, hard copies of all meeting agendas, minutes, transcripts of raw data, and field notes were provided 2. Informal meetings and telephone conversations throughout the project period 3. Email discussions were shared with one community representative, except for discussions concerning technical aspects of the project 4. Community representatives were involved in scientific conferences 5. Community representatives were interviewed to evaluate the public involvement and were involved in a workshop to generate a set of recommendations for public involvement. One community representative provided feedback
Clarke et al. 2018 [24]	Collaborate with people living with dementia as co-analysts in the coproduction (interpretation) of knowledge and re-presenting experiences within the data set	Co-analysts	Execution	Conduct	Four regionally located groups. Each group met monthly for 2 h on 4 occasions	Involvement method: secondary data analysis of four workshops with co-analysts Prior to the secondary data analysis with co-analysts, the academic researchers set up a coding framework for data analysis through two theoretical lenses. Data was analysed and a coding model was designed 1. In the first and second workshop, quotes from the data were discussed 2. In the third workshop two theoretical lenses were discussed using picture and word cards that correspond with the theories. A storyboard approach was used with two vignettes based on the data. The groups discussed how the theories were helpful to explain what was happening in the storyboard examples 3. After each workshop the academic researchers reflected on the process and how this changed their understanding of the data and theories. Metaphors used by attendees in the first three workshops were collected 4. In the fourth workshop the list of metaphors and metaphors interwoven with vignettes of two people were presented to check the academic researchers’ understanding of the metaphors 5. Metaphors drawn from the workshop were used to reinterpret the data and integrate it with theory • Workshops were facilitated by familiar local staff
Di Lorito et al. 2020 [13]	Ensuring that the study was empowering for the involved persons living with dementia (dementia appropriate) and inclusion of lived experience	Co-researchers	Translation^a^	Governance and conduct	7 one-hour interviews, preparatory meetings and training, data-analysis session	Involvement methods: co-research interviews and data-analysis workshop 1. Involvement in protocol design: lay researchers provided feedback on the protocol and how to conduct interviews with people living with dementia 2. Development of the interview schedule: discussion on interview topic guide to ensure that the interview questions were relevant, meaningful, and jargon-free for persons living with dementia 3. Preparatory and iterative training sessions: co-designing co-researcher role and tasks, collective decision that lay researchers required interview training, and making decisions on practicalities of the interviews (lay researchers had previously received training on data-analysis) 4. Co-conducting the interviews, lay researchers involved in half of the interviews: based on the concepts 'expert-by-training' and 'expert-by-experience', the academic researcher asked questions about personal beliefs and motivation, the lay researchers asked questions related to emotional support and independence. Lay and academic researchers travelled together to the participant's home to brief and debrief. The team kept personal diaries regarding methodological issues, benefits, and challenges of co-research 5. Involvement in data analysis: both academic and lay researchers read the interview transcripts, independently annotated their comments and identified themes. Academic researchers merged the annotations and themes to generate a tentative code book. Lay researchers gave feedback 6. Lay researchers were involved as co-authors in all study outputs, including publications, the evaluation protocol, presentations, and seminars 7. The team analysed the personal diaries in the home of one of the lay researchers and co-produced a Research Cycle Model for good practice in co-research
	Involving carers of persons living with dementia as co-researchers throughout the research cycle	Co-research	Preparation, execution, and translation	Governance and conduct	n/a	Model for good practice in co-research with carers of persons living with dementia (for complete model see [13]) 1. Think and plan: make plans for long-term collaboration early in the research, recruit persons based on skills etc. and with different characteristics, co-design co-researcher role 2. Prepare stage: provide iterative training based on needs and establish clear research roles 3. Gather stage: travel together to interviews, make certain that everyone is comfortable, ensure data integrity, provide emotional support to co-researcher 4. Analysis stage: ask co-researchers to independently analyse the data 5. Write and share impact stage: invite co-researchers to co-disseminate findings (i.e., co-author publications and presenting at conferences etc.)
Flavin and Sinclair, 2019 [25]	Bringing the perspectives and voices of people living with dementia and care-partners 'to the table', through advising the research team on the design, conduct, and implementation of the research	Consultation and advice	Execution and translation^a^	Governance	Quarterly meetings for 2 to 3 h in a 3-year research project	Involvement method: advisory groups Three 'supported decision-making interest groups' acted as advisory groups, supported by service provider organisations who partnered on the project 1. Meetings were facilitated, recorded and minutes with feedback provided to the broader research team 2. Providing feedback through full group and small group discussions, with consensus-based approach 3. Agreements about the confidential nature of the meeting discussions and materials • Consumers were encouraged to attend meeting with a support person • Accessible meeting materials were provided in advance (key discussion topics and questions, larger font, graphics, and space for comments, and provided in hard copy by post) and regular breaks in meeting agenda
Froggatt et al. 2015 [38]	To support researcher and residents and facilitate recruitment and conduct of interviews	Engagement in fieldwork	Execution^a^	Conduct	30 interviews, 1–5 introduction meeting, short support and follow-up meetings, 3 research team meetings	Involvement method: assistance in interviews and focus groups Research activities of the five involved persons in fieldwork: recruitment, interview facilitation, resident support, and research support 1. Recruitment: involved persons assisted in the introduction of the study to care home residents 2. Interview facilitation: (17 of 85 interviews) prior to the interview, involved persons spent time with residents to remind them about the research and interview. Involved persons supported 13 of 56 interviews with primary care staff 3. Resident support: involved persons reflected with residents how they experienced the interview, residents could ask questions and raise additional points 4. Researcher support: involved persons supported researchers during two focus groups with staff (welcoming staff, distributing information, recording, note taking) • Three project team meetings: introduction, discussion on experiences and expectations, making plans for involvement in dissemination • Site-specific preliminary meetings to introduce involved to the project • Short preparation and follow-up support meetings prior to fieldwork activities • Attendance of involved persons at final Validation event (no description)
Giebel et al., 2019 [26]	Advising and providing feedback on different aspects of the research programme	Consulting and collaborating role	Preparation and execution	Governance	Five-year research programme Small reference group: 6 biannual meetings Virtual lay advisory group: 2 email consultations	Involvement method: small reference group Follows a cyclic process of involvement: research design, selecting elements for feedback→ reference group meeting, providing feedback on impact of previous input, discussing new research ideas→ writing and distributing meeting notes; integrating feedback into research→ ethics application 1. First meeting: feedback on proposal, protocol, and research design 2. Second meeting: discussion on effective methods, services in dementia home support, and effective components 3. Third meeting: discussion on carer support services and elements that were helpful and less helpful, accessible discussion through metaphor of ingredients in a cake, consultation on acronym of the overall research programme 4. Fourth meeting: contributing to economic model of the programme through a preliminary model 5. Fifth meeting: feedback on a memory manual 6. Sixth meeting: discussion on experiences of memory clinic and hospital visits (part of the economic model group) Involvement method: virtual lay advisory group 1. Carers were asked to give feedback on the understandability of the information sheet and consent form, and they were consulted on a questionnaire 2. Consultation on understandability of five case descriptions, which will be used for research purposes with both carers and staff
Goeman et al., 2019 [27]	To share expertise and lived experiences of dementia	Advisors	Execution	Governance (and conduct in workshops)	Working group: monthly meetings during a period of 2 years Reference group: quarterly meetings during a period of 2 years	Involvement method: working group, reference group and workshop Both groups focused on the progress, aims of the study, and defined terms of reference. The terms of reference are: the purpose of the group, what was expected from members, the objectives and areas that the research would explore, a project timeline, meetings and agendas 1. Working group: guided the development of an overarching evaluation philosophy, assisted in refining the protocol for undertaking a systematic review, devised interview questions, analysed literature review outcomes and interview data 2. Reference group: supported the working group, through providing advice with regard to feasibility, applicability, policy, funding implications, and appropriate language utilisation for reporting outcomes and recommendations 3. Both groups came together for two interactive annual workshops: - First workshop: a summary of the systematic review and a list of models were provided two weeks prior to the meeting. In the meeting the group developed a framework - Second workshop: discussion on selection of summaries of interviews and mapping of models against developed framework - The results were collated by the research team and a draft report was sent to all involved persons for feedback
Gregory et al., 2020 [28]	To provide feedback on study experiences of the clinical trial, study paperwork and contribute to study planning	Research partners who give advice	Execution	Governance	All, 5 country-wide panels and ? local panels, met twice a year (except one which was held four times a year), all panels met at least twice 2 central panel meetings	Involvement method: local involved persons panels and central panel The EPAD involved persons' panels included a nested panel structure, in which local panels functioned independently and fed into a countrywide panel. Nominated members from these panels (without staff involvement) formed a central panel. The central panel fed directly into the work of the EPAD ethics workgroup and the General Assembly of the project 1. Countrywide and local panels: - Panel meeting agendas were developed by the involved persons and led by the panel chair, agendas consisted of: dementia moments (discuss recent news), update of study progress and the proof of concept trials. Additional topics and tasks were: sustainability and longevity of the project, feedback on study visit, documentation review (website, consent forms, and information videos), and communication on potential risk factors discovered through the EPAD study - Panel members contributed to scientific conferences, co-hosting a webinar on PPI, and were involved in a meeting to plan the future of the EPAD study - Staff attended to organize the logistics of the meeting, provide study updates, and answer specific questions from the panel, facilitate discussions if required and to take minutes of the meetings 2. Central panel: co-ordinated activities across the local panels and provided input for the development of the study (and future direction of the project)
Hanson et al., 2007 [29]	Discuss user needs, informing development of multimedia platform, reviewing concept of platform, provide feedback on platform and evaluation of support programme	Partnership through consultation	Execution	Conduct	9 months: weekly in first 4 months, and bi-weekly or monthly subsequently, 1 user group reviewing meeting 12-week training/support programme in three-hour weekly sessions with discussions in 5 groups, 1 focus group	Involvement methods: discussion groups, reviewing platform workshop The design process is cyclic, iterative (development, evaluation, agreement) and comprises three phases 1. Identifying user needs: - Consultation through discussion groups informed by international literature on users' needs and preferences to develop the multimedia platform - Facilitated by two project nurse facilitators 2. Early programme development: - Data from discussion sessions were used to develop the multimedia platform - The multimedia platform was reviewed by the user group by projecting the content on a large screen. Comments were subsequently integrated into the programme - A 12-week support group programme was developed by the researchers 3. Testing and refining: - In-depth interviews with people with early stage dementia (PWED) and next of kin prior to the start of support group - All involved persons (PWED and spouses) completed the 12-week support programme facilitated by the project dementia nurses and a multimedia technician - The multimedia programme was installed in all involved persons’ homes - During the 12-week programme the involved persons provided feedback in discussions and the facilitators took notes on strengths and weaknesses, feedback was again incorporated and changes made to the multimedia programme - At the end of the 12 weeks, PWED and family members took part in a focus group to evaluate the support programme
Hassan et al., 2017 [30]	Researchers were not interested in personal views, but in the views of possible future research participants. Discuss and test wearable health devices, sharing user experiences, make recommendations for future research	Advisors	Preparation	Conduct	3 workshops per group, consisting of 2 sessions over the course of 1–2 weeks and voluntary 1 week at home device testing between sessions. Informal individual sessions with people with mild cognitive impairments	Involvement methods: interactive workshops and drop-in sessions 1. Session 1: introduction to types of dementia research and questions, introduction to devices, discussion on using devices for research, opportunity to use and test devices, and invitation to borrow devices to test at home 2. Device testing period (1 week): use the device, reflect on ease of use, wearability, and barriers to use 3. Session 2: discussion on and sharing of experiences using the devices at home, considering which devices would be most suitable for different research participants in the future, reflect on user support requirements, data governance and privacy issues • Meetings were supported by discussion guides and by a series of hypothetical exemplar research scenarios. Activities were designed to be interactive, visual, and delivered at an appropriate pace for attendees to enable their involvement • At the workshops, notes were taken by assistants, and attendees recorded their ideas on flipcharts and post-it notes. After each workshop the facilitators collated the information. These reports and the overall summary report were shared with workshop attendees to check for accuracy
Mann and Hung, 2019 [31]	To offer the perspective of a person living with dementia to inform the research design and process, ensure the research benefits patients with dementia, raise awareness and change people's attitudes about patients with dementia	Advisor and co-researcher	Execution and translation	Governance and conduct	Every 4–6 weeks meeting in coffee shop over an 18-month period	Involvement method: co-research meetings in coffee shop The process included a spiral of iterative cycles of shared goals identification, joint reflection, collective action, evaluation, and modification 1. Pre-research phase 2015 - The researcher and co-researcher met and had discussions about the research proposal, purpose of research, and role of co-researcher - The researcher and co-researcher worked together on the ethics application to ensure the consent process was appropriate and meaningful for patients with dementia 2. Phase 1: Engage & Look - Environmental assessment with co-researcher and family advisors, and staff to inspire and engage staff - Meetings with co-researcher to make agreements and plans on research design and activities, conversations were guided by reflexive questions, in-between communication via email to work on documents 3. Phase 2: Think & Act - Dementia education and action activities delivered by co-researcher and staff (peer teaching) - Co-develop knowledge translation tool with co-researcher, family advisors and staff - Regular research meetings with co-researcher 4. Phase 3: Evaluate & Modify - Conference presentations by researcher and co-researcher to share research findings and receive feedback - Publications co-authored by researcher and co-researcher to contribute to literature
Poland et al., 2019 [32]	To inform and conduct research in collaboration with experts by experience	Co-applicants, advisors, and co-researchers (interviews and data analysis)	Preparation and execution	Governance and conduct	Co-applicants: n/a PPI advisory group: 7 advisory group meetings, 8 management meetings Co-researcher data collection: 12 interviews Co-research data analysis: 2 half-day workshops	Involvement methods: advisory group, co-research interviews and data-analysis workshops 1. Co-applicants: discussed research proposal, writing plain English summary, produced CV 2. Advisory group: overview of public-facing documents, support, and advice to 3 PhD projects, suggested research-focused questions, comment on newsletters and developing information 3. Co-researcher data collection: with researcher undertook 12 interviews, co-researchers led the interview with the help of the academic researcher. Provided comments on a selection of full transcripts 4. Co-research data analysis (with persons living with dementia), facilitated by the involvement coordinator, research staff and a qualitative researcher. Two half-day workshops, four people living with dementia at each workshop and four short focused activities - In two activities, the meaning of single sentences were discussed and codes developed - In the third 'interpretation' activity, co-researchers discussed short case studies - The fourth activity was a coding activity which involved selecting a theme that best related to a short phrase
Shura et al. 2010 [39]	To encourage critical and collective reflection about ideas for community improvements in long-term care, with residents as experts of long-term care life, who focused on the tasks of identifying strengths and problems in their community; to develop ways to improve community life	Visionaries	Execution	Governance and conduct	7 resident action groups, meeting 1 h per week for 4 months	Involvement method: seven unit-specific resident action groups 1. Residents are the central members of the groups, because of their lived experience and expertise regarding long-term care life. Family members and staff had supportive and collaborative roles as their experience with long-term care is qualitatively distinct from residents' views 2. Each resident group had two regular facilitators whose role was to encourage and mediate collective conversation, and to support the experts' collective process of critique and vision 3. Each resident group met on their own unit 4. Each resident group was based on interests and group dynamics that residents identified themselves; they discussed and analysed areas in need of improvement in a collective forum (examples are: lowering the bulletin boards, improving the dining experience, strengthening relationships among residents, family and staff, and opportunities for meaningful social engagement)
Stevenson and Taylor, 2019 [33]	Co-researchers were involved in identifying themes, with the aim to enhance validity by applying multiple perspectives to data analysis	Co-researcher	Execution^a^	Conduct	2-h data-analysis session	Involvement method: data-analysis session mid-way through data collection 1. Presentation to introduce the research project and clarification of co-researcher role. Sessions were attended by facilitators of service user panel. Short coffee and chat break was taken mid-session 2. Highlighters and pens were provided for the group to mark any salient words or phrases and to make notes 3. Ideas and comments were written on a flipchart as a visual reminder for the group 4. Short coffee and chat break was taken mid-session 5. Three interactive 20-min exercises, one of which focused on analysing definitions and two concerned risk communication; all discussions are guided by the connection of the data to own experiences - Quotes from interviews were presented orally and through written handouts, after which there was time for individual reflection. This was followed by a group discussion using prompts to elicit views on what the group members felt was interesting and how quotes from the interviews were connected - Excerpts from interviews with healthcare professionals and family carers were presented through role play between the facilitators and in printed handouts. Discussion through prompts. In order to remind the group of the definition of risk, the definition was presented in bold print in handouts
Swarbrick and Doors 2018 [23]	Developing a framework for co-research through reflection on own experiences in research	Co-research	Execution^a^	Governance and conduct	3 inquiry/discussion groups, each group met 8 times	Involvement method: inquiry groups The process of developing the COINED model followed four phases of co-operative inquiry. The facilitator was responsible for the exchange of ideas developed in the independent groups Phase 1: Reflection Based on prior experience with involvement in research the groups decided to explore ways of involving people living with dementia as co-researchers Phase 2: Action The three inquiry groups explored the research process through a discussion-based format. There was consensus amongst the groups and six common themes emerged Phase 3: Action—developing the model Based on the framework developed in phase 2, the inquirers developed a more elaborate description of approaches Phase 4: Reflection—the final cut Inquiry groups collectively agreed on the COINED-model
	Involving persons living with dementia as co-researchers throughout the research process	Co-research	Preparation, execution, and translation	Governance and conduct	n/a	The Co-researcher Involvement and Engagement in Dementia model, COINED-model (for the complete model see [23]) 1. Providing and developing research training and support for persons living with dementia (PLWD) and academic researchers is fundamental to successful involvement 2. Ensure ongoing consultation through the representation of PLWD in steering and advisory committees throughout the research and provide peer support 3. Involve PLWD in designing and piloting research materials 4. Involve PLWD in collecting data, such as co-conducting interviews and focus groups 5. Findings should be meaningful and translatable to practice and PLWD 6. Share research findings through creative methods to extend the discourse on dementia 7. Involve PLWD in translating research findings to practice 8. Evaluate the (self-defined) effectiveness of the involvement of PLWD as co-researchers 9. Involve PLWD in future work through identifying research priorities
Tanner, 2012 [34]	Co-researchers and interview participants establishing shared understandings and common points of reference through their shared experiences in co-conducting interviews	Co-research in interviews	Execution	Governance and conduct	3 training sessions, 8 interviews	Involvement method: co-research interviews 1. Three preparation sessions were organized to introduce the co-researchers to the study, discuss the content and structure of the interviews, and to practice interview skills. Co-researchers shared personal experiences, key points were written on a flipchart (the use of visual prompts is encouraged) and a framework for the interviews was developed. The interview framework was printed on laminated coloured cards for the co-researchers to use during the interviews 2. The role of the academic researcher during the interviews was to explain the research to participants, obtain informed consent and operate the audio recorder. The co-researchers conducted the interview with some help from the academic researcher. The academic researcher met with co-researchers in their homes to remind them about the purpose and process of the interviews and travelled together by car to the interview participants 3. Immediately after the interview, the academic and co-researcher shared thoughts on the process and content of the interview 4. After each round of interviews a meeting was organized in which academic and co-researchers discussed key themes and issues

^a^Involvement in more research phases, but these were not elaborated on

The eighteen studies introduced a variety of involvement methods, categorizable into five groups: 1) advisory groups, 2) research team meetings (both formal and informal), 3) action groups, 4) workshops, and 5) co-research in interviews. In five studies, individuals including PLWD, LTCF residents, carers, and health professionals participated in advisory/reference groups [25–27, 32], working groups [27], and panels [28]. These groups offered valuable feedback on research aspects, spanning protocols, design, questionnaires, and implementation of research. Meetings occurred at varying frequencies - monthly, quarterly, or biannually.

Two studies exemplify diverse research collaboration settings. One involving older individuals within an academic research team of five [37], and another featuring a doctoral student and a co-researcher conducting informal monthly discussions at a local coffee shop [31]. Brown et al. sought to minimize power differentials and enhance inclusivity [37], while Mann and Hung focused on benefiting people with dementia and challenging negative discourse on dementia [31].

An additional five studies employed methods involving frequent meetings, including action [35, 39], inquiry [23], and discussion groups [29, 36] In these groups, involved persons with lived experience contributed to developing a shared vision and community improvements, such as enhancing the mealtime experience in care facilities [35].

Seven studies involved individuals through workshops, often conducted over one or two sessions. These workshops contributed to generating recommendations [37], informing future e-health designs [29, 30], and ensuring diverse perspectives and lived experiences were included in data analysis [13, 24, 32, 33]. In three studies, representatives worked as co-researchers in interviews, drawing on personal experiences to enhance the interview process, making it more dementia-appropriate and enriching data collection [13, 32, 34]. Finally, one study involved representatives in the recruitment and conduct of interviews [38].

#### People involved

The number of persons involved varied from a single co-researcher [31] to 34 panel individuals providing feedback on their experiences in a clinical trial [28]. Thirteen studies focussed on PLWD: eleven involved PLWD themselves [23–27, 29–34], one exclusively focused on caregivers [13], and another one involved people without or with mild cognitive impairment, who participated in a study examining the risks of developing Alzheimer's disease [28]. Although not all articles provided descriptions of the dementia stage, available information indicated that individuals involved typically fell within the early to mid-stages of dementia [29, 30, 32–34]. Next to PLWD and carers, two studies additionally involved organizational or advocacy representatives [25, 27]. The other five studies concerned older adults living in a LTC facility. Two of them involved older residents themselves [35, 39], the other three carers, older community/client representatives or health care practitioners [36–38].

#### Roles and level of involvement

Four general roles could be identified. First, consultation and advisory roles were held by PLWD and carers [25–30, 32], where involved persons share knowledge and experiences to make suggestions [32], but the research team retained formal decision-making power [25]. Second, PLWD were involved as co-analysts in data analysis [24, 32, 33]. Co-analysts influence data analysis, but the decision-making power remained with academic researchers [24]. Third, in six studies the co-researcher role was part of the research design in which involved persons and researchers steer and conduct research together [13, 23, 31, 32, 34, 36]. Finally, two studies partnered with LTC residents [35, 39], with residents at the core of the group, and positioned as experts by experience [39]. Residents had the decision-making authority regarding how to improve life in LTC facilities [35].

#### Models for involvement in research

Only two studies designed a model for co-research with PLWD [23] or their carers [13] across all research phases. These models underscored the importance of iterative training for co-researchers [13, 23] and academic researchers [23]. Furthermore, these studies advocate involving co-researchers early on in the research process [13] and in steering committees [23]. Co-researchers can be involved in designing research materials [23], conducting interviews [13, 23], analysing data [13], and co-disseminating findings [13, 23]. Additionally, one study stressed involving PLWD in identifying (future) research priorities [23].

### Barriers, facilitators, and good practices in research phases

#### Preparation phase

Table 3 describes the barriers, facilitators, and good practices per main research phase. Lack of diversity in ethnicity and stages of dementia in the recruitment of involved persons is mentioned as a recurring barrier [26, 28, 32, 33]. The exclusion of people with cognitive impairments is partly due to gatekeepers’ and recruiters’ bias towards cognitively healthy people [28, 32]. It is stressed that researchers should refrain from making assumptions about the abilities of PLWD and ask the person what he/she is willing to do [31]. It is considered good practice to involve people regardless of cognitive abilities [23], based on skills, various personal characteristics [13] and, if possible, relevant prior experience [38].

**Table 3:** Overview of good practices, barriers, and facilitators of the included studies

Themes	Good practices	Barriers	Facilitators
**Preparation**			
Recruitment	Recruit involved persons, regardless of cognitive abilities [23], based on skills, different personal characteristics [13] and prior relevant experience [38]	Recruiting through gatekeepers (health professionals and carers) can result in the exclusion of less articulate or critical people [35], people with cognitive impairments [28], and people living with dementia [32]. A lack of identification with medical labels might lead to difficulties in recruitment [30]. Involvement of people with mild to moderate dementia does not imply representation of the experiences of people with more advanced dementia [33, 34], and involving carers might put carer’ experiences in the forefront [13, 32]. Lack of diversity, such as differences in ethnicity and religion, is an issue in PPI [26, 33]. Snowballing can result in less diversity [13]. Assuming “peerness” between co-researcher and interviewee based on sharing a dementia diagnosis and both being older persons is not enough [34]. Co-research might favour people who embrace a dementia identity and silence those who have a more self-maintaining stance [34]. The voluntary nature of PPI might imply that the skills requirements for performance of the involvement role(s) are not met [13]	Work alongside healthcare professionals and services to recruit people who are hard to reach [30]. Recruiting through existing networks and peer support groups is a good basis for building relationships [24, 38]. Involving representatives with different personalities and skills provides multiple unique perspectives and enhances data collection [13]
Role description	Explore and discuss the purpose, scope, and expectations of involvement [26, 28] at the beginning and throughout the project [29]. Provide a common, but adaptable framework [28]. Offer personal and professional development opportunities [13, 32] to make involved persons’ roles more rewarding, and support involved persons holding multiple roles [32]. Co-design the role in collaboration with involved persons, taking into account skills, preferences, and goals [13], for example using a forward planning activity sheet to enable involved persons to indicate their interests [32]	Naturally evolving roles result in a lack of clarity about expectations and involvement in irrelevant topics and tasks [37]. Integrating an involvement role in predefined governance structures was difficult and panels have not always moved in line with initial researcher expectations [28]. Access to ICT when being a co-applicant, complex language and procedures used to structure involvement roles can potentially exclude experiences of persons living with dementia [32]	The identification of clear roles, activities and tasks, and expressing expectations and values ensures that involved persons feel part of the team [38], prevents ‘tokenistic’ involvement [28] and can help the involvement process [32]. It is important to understand the involved persons' motivation for involvement to ensure personal needs are met [32]
Relationships and group dynamics	*Involved persons:* Take time to build a mutual trusting relationship [13, 23, 34], to foster freedom of expression [33] and break down social barriers [37]. Become familiar with the strengths, limitations and what the person is comfortable with in order to maximize contribution [31, 34]. Reflect on group dynamics through a hermeneutic-dialectical process [35] *Long-term care facility (LTCF) staff:* Developing a trusting relationship with LTCF staff, particularly with management and administrators, is important for involvement research in these settings to succeed [35, 36, 39]	*Involved persons:* Researchers need to stop making assumptions about the abilities of persons living with dementia [31] *LTCF staff:* LTCFs are highly hierarchical environments [35, 39]. Distrust of management and other staff towards involvement research process can lead to drop-out of organisation [36] and low staff participation [39]	*Involved persons*: Allow time for socialising [29], organize social meetings/activities [37, 38] and meetings in the home of lay researchers [13]. Pay attention to difference in roles and responsibilities [38]. Meet regularly/monthly [13, 38] even when there are no research meetings to keep involved persons motivated [34]. Travel together to interviews to brief and debrief [13, 34]. Avoid being tokenistic [13] and see the person through an appreciative lens [31]. To develop a shared voice it is important, particularly for marginalized groups, to meet in a homogenous group [35] and in small groups for involved persons s to get to know each other and feel confident to express their views [29] *LTCF staff:* Developing a shared vision is a condition for quality and validity [36]. A trusting relationship with board members and management can facilitate support of other staff [35, 36], inhibit reluctance to involvement, and prevent scheduling conflicts [39]. Develop strategies to motivate LTCF staff to become involved [39]
Training of academic and non-academic involved persons	Provide iterative coaching and training sessions [23, 31], based on emerging needs [13] or when lay researchers indicate an interest [37], to challenge the dominant view of scientific knowledge, increase reflexivity [36], ensure an appropriate skill set, establish understanding of the involvement process [28], and provide guidance and tactics to fulfil co-research role [32, 33]. Training on everyday ethics [31], how to build an equal relationship, reducing technical language and recruitment [28] should be offered to academic researchers [13, 23]	Training raises costs [28], is at odds with the principle of valuing experiential knowledge [37] and should not aim to make co-researchers into ‘expert’ researchers [32]	Training fosters confidence, skills, building rapport, a positive experience and will empower involved persons to engage, motivated, meaningfully and equally in the research process [13, 33, 38]
Financial compensation	Provide financial compensation [13, 32] for travel expenses, shopping [26], attendance at meetings [25, 27] and provide print-outs to avoid printing costs [26]	Project funding for involvement compensation was underbudgeted, involved persons only received compensations for travel expenses and accommodation [27]. Resourcing of travel expenses of involved persons was an issue [28]. Assistance with transportation should have been provided [25]	Compensation serves to acknowledge the invaluable contribution of involved persons to the research [13]
Practicalities: Time, budget and setting	Plan and budget for additional administration, time, staff, and involvement activities early on [13, 28, 33, 37]. Plan extra time for involvement activities [13, 29, 34]. The setting and timing of involvement activities matter; pay attention to: social status of location, availability of disabled access, close to facilities, and ensure that the place is familiar, comfortable, and easily accessible (close to public transport and car parking) [29–31, 34]	Limited resources restrict the desire of involved persons to meet more often [28] and hinder sustainability of initiatives [39]. Due to time constraints, representatives only supported 20% of the interviews [38]. Some forms of involvement such as co-research and action research are more resource (competences, time, and commitment of researcher) and budget intensive than other forms of involvement and may delay the research process [13, 32, 36, 39]. The physical space of involvement activities should be considered more [33], meeting in an unfamiliar place (e.g., office) could lead to stress and more distractions (e.g., less concentration) [31]	Virtual meetings are accessible regardless of location and less time consuming [26]. Empower involved persons to ensure meetings are led by them and use staff in support roles [28]. Availability of financial resources is important to implement developed ideas [35]. Develop strategies to sustain initiatives (e.g., resident councils could facilitate resident groups) [39]. Meeting in a familiar environment allows for robust involvement and enhanced concentration for people living with dementia [31]
**Execution**			
Planning of involvement design	Plan long-term collaboration with lay researchers early in the research [13]. Ensure ethics approval is in place [13] and when research ethics committees see co-analysts as research subjects, make certain no new data is gathered [24]. Make agreements on the confidential nature of research discussions and materials [25] and on what information is shared during co-research interviews [13, 32]. Ensure representation on research governance level [27], in steering and advisory groups [23]. Involving persons living with dementia as co-researchers can open up an empathic level of knowledge [23]. Invite co-researchers to analyse data independently, rather than verify the interpretations [33]	Need of an ethical framework for co-researchers which formalizes expectations, responsibilities, and confidentiality [23]. When interview guides are ethically approved prior to co-researcher involvement, this will limit the capacity of co-researchers to direct the interview [32]. The empathic bond between co-researcher and interviewee can have disadvantages, such as entrusting information in private [13],wanting to help the interviewee [32] and shared experiences might influence interviewee responses [23]. The involved persons-led design created the potential for conflicting ambitions of involved persons and researchers [28]	Early involvement increases confidence [13, 31], enables full involvement and time to build a trusting relationship [31]. Involve co-researchers in future work (e.g., identifying research priorities) in order to reposition people living with dementia at centre stage [23]. An emergent design facilitates co-ownership and ensures the research is responsive to the involved person’s experiences [35]. In relation to multisite involvement activities with central terms of reference, allow local adaptions to nurture ownership of the local group [28]
Academic culture and experiential knowledge	Lived experiences of people living with dementia should be central and guide development of research [23, 27]. Researchers should have an open mind, give up control, be open to having their traditional academic views challenged, not be dismissive of lay people’s views, step out of their comfort zone [13], value experiential knowledge and not make assumptions based on a deficit perspective [31]. Use a consensus-based approach [25] and maintain balance in joint decision making [23]	Use of academic jargon [13, 37], rapid pace of discussions [37], (subtle) power relations [36, 37], and difference between ‘academic time’ and ‘dementia time’ [24] can inhibit involved persons from expressing their views [13] and to tell about their world, and can make involved persons inclined to give socially desirable answers [37]. The dominance of the biomedical model in research decision making, the conventional social science research paradigm, the authority of research and adherence to ‘rigorous’ academic models can lead to undermining the contributions of lay researchers [13] and are a challenge to the impact of involvement, especially in relation to dementia studies where people are historically silenced [24, 32]. Including lay people in research meetings allows less space for academic talk (less efficient), expressing personal comments was not always appreciated [37] and lived experiences of lay researchers can clash with academic views and priorities [13, 23], which can be uncomfortobale for both lay researchers and academic researchers [32]. Some people might feel intimidated by the titles of academics [31] and having confidence to share personal experiences [13] or indicating a lack of understanding can be challenging [37]	Separating technical topics from general meetings [37], making a glossary of common terms to understand each other’s jargon [37], slow down to examine assumptions about dementia, reflect on the relationship and how power is shared [31], and a relational empowerment approach to facilitation, in which the facilitator acknowledges the other’s power and disempowerment, and adapts to fluctuations, could minimize power differentials [35]
Facilitation of involvement	Facilitation is very important [28, 35] and is a skill that needs practice [23]. Facilitation should include: managing and guiding involvement processes, ensuring equality of power, safeguarding the autonomy of involved persons [32], a non-directive approach, good listening skills, reflecting the words of involved persons, acceptance, positive regards for opinions [33], minimize control, embracing evolving process, appropriate risk assessments, offer professional support, be creative [23], allow time for repetition of information and clarification of tasks[29]. Inform involved persons how their input has shaped, guided, and made a difference in the research [31] at the beginning of meetings [26] and throughout the study [30]	Need for more time, opportunities for clarification [37] and more support of academic and lay researchers to ensure meaningful and effective involvement [13]. It takes time and effort to explain that scientific knowledge is not a pre-set prescription for change and needs to be contextualized, which requires reciprocal adequacy [36]	Involved persons have to be encouraged and reminded by facilitators that they are not research subjects [32, 33], are experts of lived experience [39], their knowledge and actions are valid and essential [36], that they can challenge research documents, and to note down thoughts [37]. This implies that facilitators reflect on their own actions and those of others involved [36]. Maintain a structured (i.e., set agenda), but informal and flexible meeting style, and ensure an efficient operation to encourage involved persons to express their views and enable their voices to be heard [27, 28]. Showing the impact of involvement is important to motivate involved persons s and to increase energy [31]. It facilitates a feeling of being taken seriously [28] and of intrinsic reward [32]. Active contribution of principal investigator was experienced as empowering [28]
Burden and support	Offer physical, emotional [13], and peer support [23] in relation to personal ageing and mortality [38]. Take into consideration potentially changing needs and deterioration in capabilities when involving persons living with dementia [30, 33] and help involved persons to find support when needed [29]. Identify individuals in the research team or an independent individual as a focal point of contact [28] whom involved persons can approach if they have concerns [37]. Involve experienced staff who have practical experience of working with persons living with dementia and their families [29], to offer guidance and support [30]	Some carers have dropped out due to caring difficulties [26]. Hearing or observing disturbing situations which sometimes resonate with involved persons’ own experiences can be distressing [13, 32, 38]. Involving only one person living with dementia in a group of people is experienced as intimidating and researchers showing compassion as disturbing [32]. Maximising contribution of co-researcher without overburdening him or her is a challenge [31]	Avoid too fast a pace for people who may tire easily [24, 29, 30] plan regular breaks in meetings [24, 25], be flexible regarding time frames for specific tasks and allow a ‘time-out’ when necessary [29]. Briefing and debriefing after co-researcher interviews [32] is important to provide emotional support [13]. Involve a larger team of persons to reduce the sense of burden and responsibility [37]. Involve carers or family members of persons living with dementia in the process to ensure support [25, 29, 30] and provide tailored support to avoid overburdening the carer [29]. Peer support is of importance to ensure wellbeing in the process [23], and meeting the same people at each session is beneficial for persons living with dementia and their carers [26]. Do not assume what the meaning of burden is or what support is needed [30, 31], treat people as individuals and ask the person living with dementia what he/she is willing to do or if support is needed [30, 31]
Communication/information	Establish ground rules for communication [37] and follow guidance on communicating with persons living with dementia [33]. Record and take minutes of meetings to share meeting notes with the broader research team [25] and all involved persons [26]. Share all research outputs through email or handouts [26]	Too much and irrelevant or inadequate information was shared [37], this is a problem specifically when taking into account differing abilities of involved persons [23]. Video calling-related technical problems made communication difficult [37]	To avoid an overload of information, make individual agreements on the type, amount, and format of information sent to involved persons [27, 37]. Provide summaries of key points and clearly state why the information is sent [37]
Inclusive tools	Provide pens, paper during meetings, accessible meeting materials in advance [25] and folders to organize information [37]	Use of creative visual tools should have been considered more [33]	To enable involvement of persons living with dementia use visual tools [30], such as picture word cards, storyboards and cue cards, to prompt memories [24, 34]. Modify methods and develop interactive activities to enable involvement [30, 31]. To increase visual distinctiveness, provide documents in large font [26], on coloured paper [24], with graphics and space for involved persons’ comments [25]. Provide audio-recorded notes when involved persons have difficulties with reading [26]
**Translation**			
Findings and dissemination	Findings should be meaningful to, accessible to, serve the interest of, and benefit people living with dementia [23, 31]. Use GRIPP guidelines to report on different ways of knowing and who decides [32]		Use social media and creative/visual methods to communicate research findings with the aim of increasing accessibility and extend the discourse on representation of persons living with dementia [23]
Reflection and evaluation	Determine how success of involvement will be monitored [28]. Evaluate and reflect on impact [26], effectiveness [23] and benefits of involvement [33] and on process of collaboration [31] through: writing down experiences in reflective diaries [13], using a template or paper format to guide reflection and evaluation [38] with open-ended questions [33], and joint reflection after each session and at the end of the programme [26]	There is a need for pre-set robust evaluation measures to assess impact and success of involvement [28, 33]	Reflection and evaluation can improve subsequent sessions [26], foster self-reflection and introspective learning [23], promote personal and professional development [13], and can help to address dementia-related assumptions [31]

Many studies stress the importance of building a mutual trusting relationship between involved persons and academic researchers [13, 23, 31, 33, 34, 37]. A good relationship is believed to break down social barriers [37], foster freedom of expression [33], and thereby avoiding tokenistic involvement [13]. In addition, spending time with these persons is important to become familiar with an individual’s strengths and limitations [31].

Opting for naturally evolving involvement roles was mentioned as a barrier, as this may result in conflicting expectations and irrelevant tasks [37]. A clear role description and clarification of tasks is key to balancing potentially different expectations of the involved persons and researchers [26, 28, 29, 32, 38]. When designing a role for involvement in research, good practices dictate taking into account personal skills, preferences, development goals, and motivation for involvement [13, 32]. This role should ideally be designed in collaboration with involved persons [13, 32].

The perception of providing training to involved persons is ambivalent. Studies cited that training should not aim to transform them into “pseudo-scientist” [32, 37] and that it raises the costs for involvement [28]. However, multiple scholars emphasize the importance of providing iterative training to facilitate meaningful involvement and development opportunities [13, 23, 28, 31–33, 36, 37]. Training can empower involved persons to engage in the research process equally and with confidence, with the skills to fulfil their role [13, 33, 38]. However, the implementation of training may present a potential conflict with the fundamental principle of valuing experiential knowledge [37] and should avoid the objective of transforming co-researchers into 'expert' researchers [32]. Academic researchers should also be offered training on how to facilitate meaningful involvement [13, 23, 28, 31].

Limited time and resources were mentioned as barriers to involvement that can delay the research process [13, 33, 36, 39], restrict the involvement [28] and hinder the implementation of developed ideas [39]. Financial compensation for involvement is encouraged [25–27, 32], as it acknowledges the contribution of involved persons [13]. Thus, meaningful involvement in research requires adequate funding and infrastructure to support the involvement activities [13, 28, 33, 37].

#### Execution phase

The use of academic jargon and rapid paced discussions [13, 37], power differentials, and the dominant discourse in biomedical research on what is considered “good science” can limit the impact of involvement [13, 24, 32, 36, 37]. Facilitating researchers should reflect on power differentials [35] and how decision-making power is shared [31]. Other facilitating factors are making a glossary of terms used and planning separate meetings for “technical topics” [37]. In addition, an emergent research design [35] or a design with flexible elements [28] can increase ownership in the research project and provide space for involvement to inform the research agenda [28, 35]. This requires academic researchers to value experiential knowledge and to have an open mind towards the evolving research process [13, 23, 31].

Furthermore, managing the involvement process and ensuring equity in the collaboration [13, 32, 33], facilitating researchers must encourage involved persons to voice their perspectives. This means that they sometimes need to be convinced that they are experts of lived experience [32, 33, 36, 37, 39]. To enable involvement of PLWD, the use of visual and creative tools to prompt memories can be considered [24, 30, 33, 34], as well as flexibility in relation to time frames and planning regular breaks to avoid too fast a pace for people who may tire easily [24, 25, 29, 30].

Involvement can be experienced as stressful [13, 32, 38] and caring responsibilities may interfere [26]. Tailored [29] physical and emotional support should therefore be offered [13, 23, 38] without making assumptions about the meaning of burden [30, 31]. Moreover, being the only PLWD involved in an advisory group was experienced as intimidating [25] and, ideally, a larger team of PLWD is involved to mitigate responsibilities [37]. PLWD having a focal point of contact [28, 37] and involving nurses or other staff with experience working with PLWD and their carers [29, 30] are mentioned as being beneficial. Some stress the importance of involving carers when engaging with PLWD in research [25, 29, 30].

To avoid an overload of information that is shared with the involved persons, tailoring information-sharing formats to individual preferences and abilities is essential to make communication effective [27, 37].

#### Translation

Two studies indicated a need for more robust evaluation measures to assess the effect of involvement [28, 33]. Reflection and evaluation of the involvement serves to improve the collaboration and to foster introspective learning [13, 23, 26, 31]. The included studies evaluated involvement through the use of reflective diaries [13] or a template [38] with open-ended questions [33].

Two studies postulate that findings should benefit and be accessible to PLWD [23, 31]. The use of creative tools not only enables involvement of PLWD, but can also increase accessibility of research findings and expand the present representation of PLWD [23].

## Discussion

The 18 included studies presented multiple methods for involvement in all three research phases. We found five types of involvement: advisory groups, (formal and informal) research team meetings, action groups, workshops, and co-conducting interviews. Only two studies described methods for involvement of LTC users in research. Involved persons were most often involved in consulting and advisory roles, but also as co-analysts, co-researchers, and partners. Involved persons’ roles can evolve and change over time. Especially as involved persons grow into their role, and gain confidence and knowledge of the specific research project, a more active role with shared responsibilities can become part of the research project. In addition, multiple involvement roles can be used throughout the research depending on the research phase.

Compared to the five types of involvement that we identified, other literature reviews about involvement methods for LTC users and PLWD in research also described advisory groups [10] and workshops [5, 11], and methods that were similar to research team meetings (drop-in sessions and meetings [11]). Methods for action research (action groups) and co-conducting research (interviews) were not included by these other review studies. In addition to our findings, these other reviews also described as involvement methods interviews and focus groups [5, 10] surveys [10], reader consultation [11]. Those types of methods were excluded from our study, because our definition of involvement is more strict; collecting opinions is not involvement per se, but sometimes only study participation. Moreover, compared to these previous reviews we set a high standard for transparency about the participation methods and the level of detail at which they are described.

Engaging the target group in research, particularly when collaborating with PLWD, LTC users, and carers, involves navigating unforeseen challenges [40]. This requires academic researchers to carefully balance academic research goals and expectations, and the expectations, personal circumstances and development goals related to the involved person. The aim is to maximize involvement while being attentive to the individual’s needs and avoiding a deficit perspective. Effective communication should be established, promoting respect, equality, and regular feedback between all stakeholders, including individuals living with dementia and LTCF staff. Building a mutual trusting relationship between involved persons and academic researchers through social interaction and clear communication is key to overcome barriers and ensure meaningful involvement. Inclusivity and empowerment, along with fostering an environment where diverse voices are heard, are crucial for the success of involvement in research. Our results are in line with a recent study concerning the experiences of frail older persons with involvement in research, confirming the importance of avoiding stereotypic views of ageing and frailty, building a trusting relationship, and being sensitive to older persons’ preferences and needs [41].

Furthermore, our results show that training academic researchers and involved persons is essential to develop the skills to facilitate involvement and to fulfil their role with confidence, respectively. Whilst the need for training is acknowledged by others [41, 42], there are legitimate objections to the idea of training involved persons, as the professionalization underpinning the concept of training is at odds with voicing a lay perspective [43, 44]. Furthermore, it is argued that experiential knowledge is compromised when training is structured according to the dominant professional epistemology of objectivity [45]. Therefore, training of involved persons should not focus on what researchers think they ought to know, but on what they want to learn [41].

Academic culture was frequently mentioned as a barrier to meaningful involvement. This result resonates with the wider debate related to involvement in health research which is concerned about active or “authentic involvement” being replaced with the appropriation of the patient voice as an add-on to conventional research designs [12, 46]. It is argued that such tokenistic involvement limits the involved persons’ ability to shape research outcomes [46]. To reduce tokenism requires a culture shift [13]. We believe that due to the strict definition of involvement and high transparency standard used in this review, tokenistic approaches were excluded. This may set an example for how to stimulate making this culture shift.

Furthermore, the importance of practical aspects such as funding and, by extension, the availability of time should not be underestimated. Adequate funding is necessary for compensation of involvement, but also to ensure that researchers have ample time to plan involvement activities and provide personalized support for PLWD, LTC residents and their carers. Funding bodies increasingly require involvement of the public to be part of research proposals. Yet, support in terms of financial compensation and time for the implementation of involvement in research is rarely part of funding grants [42]. In addition, whereas an emergent design could aid the impact of involvement, funders often require a pre-set research proposal in which individual components are already fixed [5, 47]. This indicates that not only do academic researchers and culture need to change, academic systems also need to be modified in order to facilitate and nurture meaningful involvement [47].

### Strengths and limitations

A key strength of this review is the inclusion of over ten scientific databases, with a reach beyond the conventional biomedical science databases often consulted in systematic reviews. Besides, we believe that we have overcome the inconsistent use of terminology of involvement in research by including also other terms used, such as participation and engagement, in our search strategy. However, there was also inconsistency in length of publications and precision of the explanation of the process of involvement. E.g., involvement in the execution phase was often elaborated on, contributions to the research proposal and co-authoring research findings were only stated and not described. This presented challenges for data extraction and analysis, as it was not always possible to identify how the target group was involved. Involvement in these research phases is therefore not fully represented in this review.

The included studies in this review, the majority of which are of high quality, provide methods for involvement of PLWD and LTC users in research and they do not explicitly attend to the effectiveness or impact of the method for involvement used. Therefore, a limitation of this review is that it cannot make any statements regarding the effectiveness of the involvement methods included. Moreover, our target population was broad, although PLWD and LTC users are largely overlapping in their care needs and share important features, this may have led to heterogeneous results. In future research, it would be interesting to interpret potential differences between involvement of PLWD, LTC users, and their carers. However, as we expected, the amount of literature included in our analyses was too limited to do so. Furthermore, whereas the broad target group is a limitation it is also a strength of our review. Limiting our search to specifically persons living in LTC facilities would have provided limited methods for involvement of persons living with dementia. Our broad target groups enabled us to learn from research projects in which people living with early staged dementia are directly involved from which we can draw lessons on the involvement of people with more advanced stages of dementia and persons living with cognitive problems who live within LTC facilities.

Since January 2021 quite some research has been published about the importance of involvement in research. Although we had quickly screened for new methods, we realise that we may have missed some involvement methods in the past years. There will be a need for a search update in the future.

### Implications for future research

Our review shows that a flexible and emergent design may help to increase involved persons' influence on and ownership in the research process. However, not all research objectives may be suitable for the implementation of an emergent design. Future research should therefore examine how aspects of a flexible emergent design can be integrated in, e.g., clinical research without compromising the validity of research outcomes.

Alzheimer Europe has called for the direct involvement of persons living with dementia in research [48]. In addition, Swarbrick et al. (this review) advise to involve persons regardless of their cognitive abilities [23]. These statements question the involvement of proxies, such as carers, professional caregivers and others involved in the care of PLWD. While PLWD and persons with other cognitive problems constitute a significant group within residential and nursing homes [7], none of the studies included in this review have provided methods to directly involve persons with more advanced stages of dementia. This raises the question if research methods should be adapted to allow those with more advanced stages of dementia to be involved themselves or if, concerning the progressive nature of the disease, it is more appropriate to involve proxies. And secondly who should these proxies be? Those that care for and live with persons with an advanced stage of dementia, or for example a person living with an early stage of dementia to represent the voices of persons with more advanced stages of dementia [31]?

Future research should adopt our example for stricter requirements for involvement and transparency about the involvement methods used. This will reduce tokenistic involvement and further promote the culture shift towards meaningful involvement. In addition, future research should assess the impact of the involvement methods that are described in this review. One of the first instruments that that may be used to do so in varying healthcare settings is the Public and Patient Engagement Evaluation Tool (PPEET) [49]. Moreover, scholars in this review stress, and we agree with this, that future research is needed on the involvement of persons with more advanced stages of dementia to ensure their voices are not excluded from research [33, 34].

## Conclusions

This review provides an overview of the existing methods used to actively involve PLWD, LTC users, and carers in scientific research. Our findings show that their involvement is feasible throughout all research phases. We have identified five different methods for involvement, four different roles, and two models for co-research. Our results suggest that planning enough time for involving PLWD, LTC users, and carers in research, is important to ensure that researchers have time to build a trusting relationship and meet their personal needs and preferences. In addition, researchers are advised not to presume the meaning of burden and to avoid a deficit perspective. A flexible or emergent design could aid involved persons’ ownership in the research process.

### Supplementary Information

**Supplementary Material 1.**

## Data Availability

The full search strategy is provided in supplement 1. The data extraction form can be provided by the corresponding author on reasonable request.

## Abbreviations

CASP: Critical Appraisal Skills Programme
GRIPP2-LF: Guidance for Reporting Involvement of Patients and the Public, long form version 2
LTC: Long-term care
PLWD: Persons living with dementia

## References

[1] Domecq JP, Prutsky G, Elraiyah T (2014). Patient engagement in research: a systematic review. BMC Health Serv Res.

[2] Brett J, Staniszewska S, Mockford C (2014). A systematic review of the impact of patient and public involvement on service users, researchers and communities. Patient-Patient-Centered Outcomes Res.

[3] Staley K. Exploring impact: Public involvement in NHS, public health and social care research. 2009. Available from: https://www.invo.org.uk/wp-content/uploads/2011/11/Involve_Exploring_Impactfinal28.10.09.pdf. Accessed 17 Jan 2024

[4] Ashcroft J, Wykes T, Taylor J (2016). Impact on the individual: what do patients and carers gain, lose and expect from being involved in research?. J Ment Health.

[5] Backhouse T, Kenkmann A, Lane K (2016). Older care-home residents as collaborators or advisors in research: a systematic review. Age Ageing.

[6] Bendien E, Groot B, Abma T. Circles of impacts within and beyond participatory action research with older people. Ageing Soc 2020:1–21. 10.1017/S0144686X20001336.

[7] Lepore MED, Meyer J, Igarashi A. How long-term care quality assurance measures address dementia in Australia, England, Japan, and the United States. Ageing Health Res 2021;1(2). 10.1016/j.ahr.2021.100013.

[8] Freedman VA, Spillman BC (2014). Disability and care needs among older Americans. Milbank Q.

[9] Bethell J, Commisso E, Rostad HM (2018). Patient engagement in research related to dementia: a scoping review. Dementia.

[10] Miah J, Dawes P, Edwards S (2019). Patient and public involvement in dementia research in the European Union: a scoping review. BMC Geriatr.

[11] Schilling I, Gerhardus A (2017). Methods for involving older people in health research—a review of the literature. Int J Environ Res Public Health.

[12] Andersson N (2018). Participatory research—A modernizing science for primary health care. J Gen Fam Med.

[13] Di Lorito C, Godfrey M, Dunlop M (2020). Adding to the knowledge on patient and public involvement: reflections from an experience of co-research with carers of people with dementia. Health Expect.

[14] Islam S, Small N (2020). An annotated and critical glossary of the terminology of inclusion in healthcare and health research. Res Involve Engage.

[15] Rose D (2014). Patient and public involvement in health research: Ethical imperative and/or radical challenge?. J Health Psychol.

[16] NIHR. Briefing notes for researchers: public involvement in NHS, health and social care research. https://www.nihr.ac.uk/documents/briefing-notes-for-researchers-public-involvement-in-nhs-health-and-social-care-research/27371#Involvement. Accessed 12 Jan 2023.

[17] Critical Appraisal Skills Programme. CASP Qualitative Checklist. 2018. https://casp-uk.net/images/checklist/documents/CASP-Qualitative-Studies-Checklist/CASP-Qualitative-Checklist-2018_fillable_form.pdf. Accessed 12 Jan 2023.

[18] Staniszewska S, Brett J, Simera I (2017). GRIPP2 reporting checklists: tools to improve reporting of patient and public involvement in research. Res Involv Engagem.

[19] Shippee ND, Domecq Garces JP, Prutsky Lopez GJ (2015). Patient and service user engagement in research: a systematic review and synthesized framework. Health Expect.

[20] Thomas J, Harden A (2008). Methods for the thematic synthesis of qualitative research in systematic reviews. BMC Med Res Methodol.

[21] Saldana JM (2015). The coding manual for qualitative researchers.

[22] Page MJ, McKenzie JE, Bossuyt PM, The PRISMA (2020). statement: an updated guideline for reporting systematic reviews. BMJ.

[23] Swarbrick C, Doors O, Keady J, Hydén L-C, Johnson A (2018). Developing the co-researcher involvement and engagement in dementia model (COINED): A co-operative inquiry. Social research methods in dementia studies: Inclusion and innovation.

[24] Clarke CL, Wilkinson H, Watson J (2018). A seat around the table: participatory data analysis with people living with dementia. Qual Health Res.

[25] Flavin T, Sinclair C (2019). Reflections on involving people living with dementia in research in the Australian context. Australas J Ageing.

[26] Giebel C, Roe B, Hodgson A (2019). Effective public involvement in the HoST-D programme for dementia home care support: From proposal and design to methods of data collection (innovative practice). Dementia (London).

[27] Goeman DP, Corlis M, Swaffer K (2019). Partnering with people with dementia and their care partners, aged care service experts, policymakers and academics: a co-design process. Australas J Ageing.

[28] Gregory S, Bunnik EM, Callado AB (2020). Involving research participants in a pan-European research initiative: the EPAD participant panel experience. Res Involv Engagem.

[29] Hanson E, Magnusson L, Arvidsson H (2007). Working together with persons with early stage dementia and their family members to design a user-friendly technology-based support service. Dementia.

[30] Hassan L, Swarbrick C, Sanders C (2017). Tea, talk and technology: patient and public involvement to improve connected health 'wearables' research in dementia. Res Involv Engagem.

[31] Mann J, Hung L (2019). Co-research with people living with dementia for change. Action Research.

[32] Poland F, Charlesworth G, Leung P (2019). Embedding patient and public involvement: managing tacit and explicit expectations. Health Expect.

[33] Stevenson M, Taylor BJ (2019). Involving individuals with dementia as co-researchers in analysis of findings from a qualitative study. Dementia (London).

[34] Tanner D (2012). Co-research with older people with dementia: experience and reflections. J Ment Health.

[35] Baur V, Abma T (2012). 'The Taste Buddies': participation and empowerment in a residential home for older people. Ageing Soc.

[36] Beukema L, Valkenburg B (2007). Demand-driven elderly care in the Netherlands. Action Research.

[37] Brown LJE, Dickinson T, Smith S (2018). Openness, inclusion and transparency in the practice of public involvement in research: a reflective exercise to develop best practice recommendations. Health Expect.

[38] Froggatt K, Goodman C, Morbey H (2016). Public involvement in research within care homes: benefits and challenges in the APPROACH study. Health Expect.

[39] Shura R, Siders RA, Dannefer D (2011). Culture change in long-term care: participatory action research and the role of the resident. Gerontologist.

[40] Cook T (2020). Participatory research: Its meaning and messiness. Beleidsonderzoek Online.

[41] Haak M, Ivanoff S, Barenfeld E (2021). Research as an essentiality beyond one’s own competence: an interview study on frail older people's view of research. Res Involv Engage.

[42] Chamberlain SA, Gruneir A, Keefe JM (2021). Evolving partnerships: engagement methods in an established health services research team. Res Involve Engage.

[43] Bélisle-Pipon J-C, Rouleau G, Birko S (2018). Early-career researchers’ views on ethical dimensions of patient engagement in research. BMC Med Ethics.

[44] Ives J, Damery S, Redwod S (2013). PPI, paradoxes and Plato: who's sailing the ship?. J Med Ethics.

[45] De Graaff M, Stoopendaal A, Leistikow I (2019). Transforming clients into experts-by-experience: a pilot in client participation in Dutch long-term elderly care homes inspectorate supervision. Health Policy.

[46] Cook T. Where Participatory Approaches Meet Pragmatism in Funded (Health) Research: The Challenge of Finding Meaningful Spaces. Forum Qualitative Sozialforschung Forum: Qualitative Social Research. 2012;13(1). 10.17169/fqs-13.1.1783.

[47] Paylor J, McKevitt C (2019). The possibilities and limits of “co-producing” research. Front Sociol.

[48] Gove D, Diaz-Ponce A, Georges J (2018). Alzheimer Europe's position on involving people with dementia in research through PPI (patient and public involvement). Aging Ment Health.

[49] Abelson J, Li K, Wilson G (2016). Supporting quality public and patient engagement in health system organizations: development and usability testing of the Public and Patient Engagement Evaluation Tool. Health Expect.

