# Supplementary material for: Active involvement in scientific research of persons living with dementia and long-term care users: a systematic review of existing methods with a specific focus on good practices, facilitators and barriers of involvement
Source: BMC Geriatr. 2024 Apr 9;24:324. doi: 10.1186/s12877-024-04877-7 (PMC11003093; doi:10.1186/s12877-024-04877-7)
Supplement: Supplementary file 1 — Supplementary Material 1. [file 12877_2024_4877_MOESM1_ESM.docx]

Supplement 1

Participation in scientific research of persons with dementia and residents in long-term care: a systematic review of existing methods

1. **PubMed**

(("Patient Participation"[Mesh] OR "patient participation"[tw] OR "client participation"[tw] OR "consumer participation"[tw] OR "user participation"[tw] OR "carer participation"[tw] OR "caregiver participation"[tw] OR "public participation"[tw] OR "citizen participation"[tw] OR "stakeholder participation"[tw] OR "representative participation"[tw] OR "relative participation"[tw] OR "family participation"[tw] OR "lay participation"[tw] OR "resident participation"[tw] OR "patient involvement"[tw] OR "client involvement"[tw] OR "consumer involvement"[tw] OR "user involvement"[tw] OR "carer involvement"[tw] OR "caregiver involvement"[tw] OR "public involvement"[tw] OR "citizen involvement"[tw] OR "stakeholder involvement"[tw] OR "representative involvement"[tw] OR "relative involvement"[tw] OR "family involvement"[tw] OR "lay involvement"[tw] OR "resident involvement"[tw] OR "patient engagement"[tw] OR "client engagement"[tw] OR "consumer engagement"[tw] OR "user engagement"[tw] OR "carer engagement"[tw] OR "caregiver engagement"[tw] OR "public engagement"[tw] OR "citizen engagement"[tw] OR "stakeholder engagement"[tw] OR "representative engagement"[tw] OR "relative engagement"[tw] OR "family engagement"[tw] OR "lay engagement"[tw] OR "resident engagement"[tw] OR "patient collaboration"[tw] OR "client collaboration"[tw] OR "consumer collaboration"[tw] OR "user collaboration"[tw] OR "carer collaboration"[tw] OR "caregiver collaboration"[tw] OR "public collaboration"[tw] OR "citizen collaboration"[tw] OR "stakeholder collaboration"[tw] OR "representative collaboration"[tw] OR "relative collaboration"[tw] OR "family collaboration"[tw] OR "lay collaboration"[tw] OR "resident collaboration"[tw] OR "patient partnership"[tw] OR "client partnership"[tw] OR "consumer partnership"[tw] OR "user partnership"[tw] OR "carer partnership"[tw] OR "caregiver partnership"[tw] OR "public partnership"[tw] OR "citizen partnership"[tw] OR "stakeholder partnership"[tw] OR "representative partnership"[tw] OR "relative partnership"[tw] OR "family partnership"[tw] OR "lay partnership"[tw] OR "resident partnership"[tw] OR "patient empowerment"[tw] OR "client empowerment"[tw] OR "consumer empowerment"[tw] OR "user empowerment"[tw] OR "carer empowerment"[tw] OR "caregiver empowerment"[tw] OR "public empowerment"[tw] OR "citizen empowerment"[tw] OR "stakeholder empowerment"[tw] OR "representative empowerment"[tw] OR "relative empowerment"[tw] OR "family empowerment"[tw] OR "lay empowerment"[tw] OR "resident empowerment"[tw] OR "patient consultation"[tw] OR "client consultation"[tw] OR "consumer consultation"[tw] OR "user consultation"[tw] OR "carer consultation"[tw] OR "caregiver consultation"[tw] OR "public consultation"[tw] OR "citizen consultation"[tw] OR "stakeholder consultation"[tw] OR "representative consultation"[tw] OR "relative consultation"[tw] OR "family consultation"[tw] OR "lay consultation"[tw] OR "resident consultation"[tw] OR "Patient Activation"[tw] OR "Patient Activations"[tw] OR co-research*[tw] OR coresearch*[tw]) AND ("Residential Facilities"[Mesh] OR "Residential Facilities"[tw] OR "Residential Facility"[tw] OR "Nursing Homes"[mesh] OR "Nursing Homes"[tw] OR "Nursing Home"[tw] OR "Institutionalization"[mesh] OR "Institutionalization"[tw] OR "Institutionalisation"[tw] OR "Long-Term Care"[mesh] OR "Long-Term Care"[tw] OR "Home resident"[tw] OR "Home residents"[tw] OR "Dementia"[mesh] OR "dementia"[tw] OR dement*[tw] OR alzheimer*[tw] OR "Mental healthcare"[tw] OR "Mental health care"[tw] OR "Mental Health Services"[Mesh] OR "Mental Health Services"[tw] OR "Mental Health Service"[tw] OR "Disabled Persons"[Mesh] OR "Disabled"[tw] OR Cognitive impair*[tw] OR "Cognitive Dysfunction"[Mesh]) AND ("Research"[majr] OR "Biomedical research"[majr] OR "Medical research"[ti] OR "Biomedical research"[ti] OR "Care research"[ti] OR "Healthcare research"[ti] OR "Scientific research"[ti] OR "Research agenda"[ti] OR "Research agendas"[ti] OR "research priorities"[ti] OR "research priority"[ti] OR "Research programs"[ti] OR "Research program"[ti] OR "Research programmes"[ti] OR "Research programme"[ti] OR "Research methods"[ti] OR "Research method"[ti] OR "Research questions"[ti] OR "Research question"[ti] OR "Participatory research"[ti] OR co research*[ti] OR coresearch*[ti] OR "Behavioral Research"[ti] OR "Health Services Research"[ti] OR "Community-Based Participatory Research"[ti] OR "Comparative Effectiveness Research"[ti] OR "Global Burden of Disease"[ti] OR "Health Care Survey"[ti] OR "Health Care Surveys"[ti] OR "Health Impact Assessment"[ti] OR "Health Services Needs and Demand"[ti] OR "Needs Assessment"[ti] OR "Organizational Case Studies"[ti] OR "Organizational Case Study"[ti] OR "Patient Reported Outcome Measure"[ti] OR "Patient Reported Outcome Measures"[ti] OR "Human Experimentation"[ti] OR "Nursing Research"[ti] OR "Outcome Assessment"[ti] OR "Treatment Outcome"[ti] OR "Pharmaceutical Research"[ti] OR "Pharmacy Research"[ti] OR "Rehabilitation Research"[ti] OR "Community-Based Participatory Research"[ti] OR "Community-Based Participatory Research"[majr] OR "Empirical Research"[majr] OR "Grounded Theory"[majr] OR "Qualitative Research"[majr] OR "Interdisciplinary Research"[majr] OR "Operations Research"[majr] OR "Peer Review, Research"[majr] OR "Public Health Systems Research"[majr] OR "Research Design"[majr] OR "Research Report"[majr] OR "Empirical Research"[ti] OR "Grounded Theory"[ti] OR "Qualitative Research"[ti] OR "Interdisciplinary Research"[ti] OR "Operations Research"[ti] OR "Peer Review Research"[ti] OR "Public Health Systems Research"[ti] OR "Research Design"[ti] OR "Research Report"[ti] OR "Research Subjects"[majr] OR "Research Subjects"[ti] OR "Research Subject"[ti] OR "Participatory Research"[ti] **OR "research"[ti]**) AND (Reform*[tw] OR Develop*[tw] OR Change*[tw] OR Design*[tw] OR Redesign*[tw] OR "Conceptualization"[tw] OR "Conceptualisation"[tw] OR concept*[tw] OR Model*[tw] OR Method*[tw] OR "strategy"[tw] OR "strategies"[tw] OR strateg*[tw]))

1. **MEDLINE**

((exp "Patient Participation"/ OR "patient participation".mp OR "client participation".mp OR "consumer participation".mp OR "user participation".mp OR "carer participation".mp OR "caregiver participation".mp OR "public participation".mp OR "citizen participation".mp OR "stakeholder participation".mp OR "representative participation".mp OR "relative participation".mp OR "family participation".mp OR "lay participation".mp OR "resident participation".mp OR "patient involvement".mp OR "client involvement".mp OR "consumer involvement".mp OR "user involvement".mp OR "carer involvement".mp OR "caregiver involvement".mp OR "public involvement".mp OR "citizen involvement".mp OR "stakeholder involvement".mp OR "representative involvement".mp OR "relative involvement".mp OR "family involvement".mp OR "lay involvement".mp OR "resident involvement".mp OR "patient engagement".mp OR "client engagement".mp OR "consumer engagement".mp OR "user engagement".mp OR "carer engagement".mp OR "caregiver engagement".mp OR "public engagement".mp OR "citizen engagement".mp OR "stakeholder engagement".mp OR "representative engagement".mp OR "relative engagement".mp OR "family engagement".mp OR "lay engagement".mp OR "resident engagement".mp OR "patient collaboration".mp OR "client collaboration".mp OR "consumer collaboration".mp OR "user collaboration".mp OR "carer collaboration".mp OR "caregiver collaboration".mp OR "public collaboration".mp OR "citizen collaboration".mp OR "stakeholder collaboration".mp OR "representative collaboration".mp OR "relative collaboration".mp OR "family collaboration".mp OR "lay collaboration".mp OR "resident collaboration".mp OR "patient partnership".mp OR "client partnership".mp OR "consumer partnership".mp OR "user partnership".mp OR "carer partnership".mp OR "caregiver partnership".mp OR "public partnership".mp OR "citizen partnership".mp OR "stakeholder partnership".mp OR "representative partnership".mp OR "relative partnership".mp OR "family partnership".mp OR "lay partnership".mp OR "resident partnership".mp OR "patient empowerment".mp OR "client empowerment".mp OR "consumer empowerment".mp OR "user empowerment".mp OR "carer empowerment".mp OR "caregiver empowerment".mp OR "public empowerment".mp OR "citizen empowerment".mp OR "stakeholder empowerment".mp OR "representative empowerment".mp OR "relative empowerment".mp OR "family empowerment".mp OR "lay empowerment".mp OR "resident empowerment".mp OR "patient consultation".mp OR "client consultation".mp OR "consumer consultation".mp OR "user consultation".mp OR "carer consultation".mp OR "caregiver consultation".mp OR "public consultation".mp OR "citizen consultation".mp OR "stakeholder consultation".mp OR "representative consultation".mp OR "relative consultation".mp OR "family consultation".mp OR "lay consultation".mp OR "resident consultation".mp OR "Patient Activation".mp OR "Patient Activations".mp OR co-research*.mp OR coresearch*.mp) AND (exp "Residential Facilities"/ OR "Residential Facilities".mp OR "Residential Facility".mp OR exp "Nursing Homes"/ OR "Nursing Homes".mp OR "Nursing Home".mp OR exp "Institutionalization"/ OR "Institutionalization".mp OR "Institutionalisation".mp OR exp "Long-Term Care"/ OR "Long-Term Care".mp OR "Home resident".mp OR "Home residents".mp OR exp "Dementia"/ OR "dementia".mp OR dement*.mp OR alzheimer*.mp OR "Mental healthcare".mp OR "Mental health care".mp OR exp "Mental Health Services"/ OR "Mental Health Services".mp OR "Mental Health Service".mp OR exp "Disabled Persons"/ OR "Disabled".mp OR Cognitive impair*.mp OR exp "Cognitive Dysfunction"/) AND (exp *"Research"/ OR exp *"Biomedical research"/ OR "Medical research".ti OR "Biomedical research".ti OR "Care research".ti OR "Healthcare research".ti OR "Scientific research".ti OR "Research agenda".ti OR "Research agendas".ti OR "research priorities".ti OR "research priority".ti OR "Research programs".ti OR "Research program".ti OR "Research programmes".ti OR "Research programme".ti OR "Research methods".ti OR "Research method".ti OR "Research questions".ti OR "Research question".ti OR "Participatory research".ti OR "co research*".ti OR coresearch*.ti OR "Behavioral Research".ti OR "Health Services Research".ti OR "Community-Based Participatory Research".ti OR "Comparative Effectiveness Research".ti OR "Global Burden of Disease".ti OR "Health Care Survey".ti OR "Health Care Surveys".ti OR "Health Impact Assessment".ti OR "Health Services Needs and Demand".ti OR "Needs Assessment".ti OR "Organizational Case Studies".ti OR "Organizational Case Study".ti OR "Patient Reported Outcome Measure".ti OR "Patient Reported Outcome Measures".ti OR "Human Experimentation".ti OR "Nursing Research".ti OR "Outcome Assessment".ti OR "Treatment Outcome".ti OR "Pharmaceutical Research".ti OR "Pharmacy Research".ti OR "Rehabilitation Research".ti OR "Community-Based Participatory Research".ti OR exp *"Community-Based Participatory Research"/ OR exp *"Empirical Research"/ OR exp *"Grounded Theory"/ OR exp *"Qualitative Research"/ OR exp *"Interdisciplinary Research"/ OR exp *"Operations Research"/ OR exp *"Peer Review, Research"/ OR exp *"Public Health Systems Research"/ OR exp *"Research Design"/ OR exp *"Research Report"/ OR "Empirical Research".ti OR "Grounded Theory".ti OR "Qualitative Research".ti OR "Interdisciplinary Research".ti OR "Operations Research".ti OR "Peer Review, Research".ti OR "Public Health Systems Research".ti OR "Research Design".ti OR "Research Report".ti OR exp *"Research Subjects"/ OR "Research Subjects".ti OR "Research Subject".ti OR "Participatory Research".ti **OR "research".ti**) AND (Reform*.mp OR Develop*.mp OR Change*.mp OR Design*.mp OR Redesign*.mp OR "Conceptualization".mp OR "Conceptualisation".mp OR concept*.mp OR Model*.mp OR Method*.mp OR "strategy".mp OR "strategies".mp OR strateg*.mp))

1. **Embase**

(("patient participation"/ OR "patient participation".mp OR "client participation".mp OR "consumer participation".mp OR "user participation".mp OR "carer participation".mp OR "caregiver participation".mp OR "public participation".mp OR "citizen participation".mp OR "stakeholder participation".mp OR "representative participation".mp OR "relative participation".mp OR "family participation".mp OR "lay participation".mp OR "resident participation".mp OR "patient involvement".mp OR "client involvement".mp OR "consumer involvement".mp OR "user involvement".mp OR "carer involvement".mp OR "caregiver involvement".mp OR "public involvement".mp OR "citizen involvement".mp OR "stakeholder involvement".mp OR "representative involvement".mp OR "relative involvement".mp OR "family involvement".mp OR "lay involvement".mp OR "resident involvement".mp OR "patient engagement".mp OR "client engagement".mp OR "consumer engagement".mp OR "user engagement".mp OR "carer engagement".mp OR "caregiver engagement".mp OR "public engagement".mp OR "citizen engagement".mp OR "stakeholder engagement".mp OR "representative engagement".mp OR "relative engagement".mp OR "family engagement".mp OR "lay engagement".mp OR "resident engagement".mp OR "patient collaboration".mp OR "client collaboration".mp OR "consumer collaboration".mp OR "user collaboration".mp OR "carer collaboration".mp OR "caregiver collaboration".mp OR "public collaboration".mp OR "citizen collaboration".mp OR "stakeholder collaboration".mp OR "representative collaboration".mp OR "relative collaboration".mp OR "family collaboration".mp OR "lay collaboration".mp OR "resident collaboration".mp OR "patient partnership".mp OR "client partnership".mp OR "consumer partnership".mp OR "user partnership".mp OR "carer partnership".mp OR "caregiver partnership".mp OR "public partnership".mp OR "citizen partnership".mp OR "stakeholder partnership".mp OR "representative partnership".mp OR "relative partnership".mp OR "family partnership".mp OR "lay partnership".mp OR "resident partnership".mp OR "patient empowerment".mp OR "client empowerment".mp OR "consumer empowerment".mp OR "user empowerment".mp OR "carer empowerment".mp OR "caregiver empowerment".mp OR "public empowerment".mp OR "citizen empowerment".mp OR "stakeholder empowerment".mp OR "representative empowerment".mp OR "relative empowerment".mp OR "family empowerment".mp OR "lay empowerment".mp OR "resident empowerment".mp OR "patient consultation".mp OR "client consultation".mp OR "consumer consultation".mp OR "user consultation".mp OR "carer consultation".mp OR "caregiver consultation".mp OR "public consultation".mp OR "citizen consultation".mp OR "stakeholder consultation".mp OR "representative consultation".mp OR "relative consultation".mp OR "family consultation".mp OR "lay consultation".mp OR "resident consultation".mp OR "Patient Activation".mp OR "Patient Activations".mp OR "co-research*".mp OR coresearch*.mp) AND ("Residential Home"/ OR "Residential Facilities".mp OR "Residential Facility".mp OR "Nursing Home"/ OR "Nursing Homes".mp OR "Nursing Home".mp OR "Institutionalization"/ OR "institutional care"/ OR "Institutionalization".mp OR "Institutionalisation".mp OR "Long Term Care"/ OR "Long-Term Care".mp OR "nursing home patient"/ OR "Home resident".mp OR "Home residents".mp OR exp "Dementia"/ OR "dementia".mp OR dement*.mp OR alzheimer*.mp OR exp "Mental health care"/ OR "Mental healthcare".mp OR "Mental health care".mp OR exp "Mental Health Service"/ OR "Mental Health Services".mp OR "Mental Health Service".mp OR exp "Disabled Person"/ OR "Disabled".mp OR "Cognitive impair*".mp OR exp "Cognitive Defect"/) AND (exp *"Research"/ OR exp *"Medical research"/ OR "Medical research".ti OR "Biomedical research".ti OR "Care research".ti OR "Healthcare research".ti OR "Scientific research".ti OR "Research agenda".ti OR "Research agendas".ti OR "research priorities".ti OR "research priority".ti OR "Research programs".ti OR "Research program".ti OR "Research programmes".ti OR "Research programme".ti OR "Research methods".ti OR "Research method".ti OR "Research questions".ti OR "Research question".ti OR "Participatory research".ti OR "co research*".ti OR coresearch*.ti OR "Behavioral Research".ti OR "Health Services Research".ti OR "Community-Based Participatory Research".ti OR "Comparative Effectiveness Research".ti OR "Global Burden of Disease".ti OR "Health Care Survey".ti OR "Health Care Surveys".ti OR "Health Impact Assessment".ti OR "Health Services Needs and Demand".ti OR "Needs Assessment".ti OR "Organizational Case Studies".ti OR "Organizational Case Study".ti OR "Patient Reported Outcome Measure".ti OR "Patient Reported Outcome Measures".ti OR "Human Experimentation".ti OR "Nursing Research".ti OR "Outcome Assessment".ti OR "Treatment Outcome".ti OR "Pharmaceutical Research".ti OR "Pharmacy Research".ti OR "Rehabilitation Research".ti OR "Community-Based Participatory Research".ti OR *"Participatory Research"/ OR *"Empirical Research"/ OR exp *"Grounded Theory"/ OR exp *"Qualitative Research"/ OR exp *"Interdisciplinary Research"/ OR exp *"Operations Research"/ OR exp *"Public Health Systems Research"/ OR ***"Methodology"**/ OR "Empirical Research".ti OR "Grounded Theory".ti OR "Qualitative Research".ti OR "Interdisciplinary Research".ti OR "Operations Research".ti OR "Peer Review, Research".ti OR "Public Health Systems Research".ti OR "Research Design".ti OR "Research Report".ti OR *"Research Subject"/ OR "Research Subjects".ti OR "Research Subject".ti OR "Participatory Research".ti **OR "research".ti**) AND (Reform*.mp OR Develop*.mp OR Change*.mp OR Design*.mp OR Redesign*.mp OR "Conceptualization".mp OR "Conceptualisation".mp OR concept*.mp OR Model*.mp OR Method*.mp OR "strategy".mp OR "strategies".mp OR strateg*.mp))

NOT (conference review or conference abstract).pt

1. **Emcare**

(("patient participation"/ OR "patient participation".mp OR "client participation".mp OR "consumer participation".mp OR "user participation".mp OR "carer participation".mp OR "caregiver participation".mp OR "public participation".mp OR "citizen participation".mp OR "stakeholder participation".mp OR "representative participation".mp OR "relative participation".mp OR "family participation".mp OR "lay participation".mp OR "resident participation".mp OR "patient involvement".mp OR "client involvement".mp OR "consumer involvement".mp OR "user involvement".mp OR "carer involvement".mp OR "caregiver involvement".mp OR "public involvement".mp OR "citizen involvement".mp OR "stakeholder involvement".mp OR "representative involvement".mp OR "relative involvement".mp OR "family involvement".mp OR "lay involvement".mp OR "resident involvement".mp OR "patient engagement".mp OR "client engagement".mp OR "consumer engagement".mp OR "user engagement".mp OR "carer engagement".mp OR "caregiver engagement".mp OR "public engagement".mp OR "citizen engagement".mp OR "stakeholder engagement".mp OR "representative engagement".mp OR "relative engagement".mp OR "family engagement".mp OR "lay engagement".mp OR "resident engagement".mp OR "patient collaboration".mp OR "client collaboration".mp OR "consumer collaboration".mp OR "user collaboration".mp OR "carer collaboration".mp OR "caregiver collaboration".mp OR "public collaboration".mp OR "citizen collaboration".mp OR "stakeholder collaboration".mp OR "representative collaboration".mp OR "relative collaboration".mp OR "family collaboration".mp OR "lay collaboration".mp OR "resident collaboration".mp OR "patient partnership".mp OR "client partnership".mp OR "consumer partnership".mp OR "user partnership".mp OR "carer partnership".mp OR "caregiver partnership".mp OR "public partnership".mp OR "citizen partnership".mp OR "stakeholder partnership".mp OR "representative partnership".mp OR "relative partnership".mp OR "family partnership".mp OR "lay partnership".mp OR "resident partnership".mp OR "patient empowerment".mp OR "client empowerment".mp OR "consumer empowerment".mp OR "user empowerment".mp OR "carer empowerment".mp OR "caregiver empowerment".mp OR "public empowerment".mp OR "citizen empowerment".mp OR "stakeholder empowerment".mp OR "representative empowerment".mp OR "relative empowerment".mp OR "family empowerment".mp OR "lay empowerment".mp OR "resident empowerment".mp OR "patient consultation".mp OR "client consultation".mp OR "consumer consultation".mp OR "user consultation".mp OR "carer consultation".mp OR "caregiver consultation".mp OR "public consultation".mp OR "citizen consultation".mp OR "stakeholder consultation".mp OR "representative consultation".mp OR "relative consultation".mp OR "family consultation".mp OR "lay consultation".mp OR "resident consultation".mp OR "Patient Activation".mp OR "Patient Activations".mp OR "co-research*".mp OR coresearch*.mp) AND ("Residential Home"/ OR "Residential Facilities".mp OR "Residential Facility".mp OR "Nursing Home"/ OR "Nursing Homes".mp OR "Nursing Home".mp OR "Institutionalization"/ OR "institutional care"/ OR "Institutionalization".mp OR "Institutionalisation".mp OR "Long Term Care"/ OR "Long-Term Care".mp OR "nursing home patient"/ OR "Home resident".mp OR "Home residents".mp OR exp "Dementia"/ OR "dementia".mp OR dement*.mp OR alzheimer*.mp OR exp "Mental health care"/ OR "Mental healthcare".mp OR "Mental health care".mp OR exp "Mental Health Service"/ OR "Mental Health Services".mp OR "Mental Health Service".mp OR exp "Disabled Person"/ OR "Disabled".mp OR "Cognitive impair*".mp OR exp "Cognitive Defect"/) AND (exp *"Research"/ OR exp *"Medical research"/ OR "Medical research".ti OR "Biomedical research".ti OR "Care research".ti OR "Healthcare research".ti OR "Scientific research".ti OR "Research agenda".ti OR "Research agendas".ti OR "research priorities".ti OR "research priority".ti OR "Research programs".ti OR "Research program".ti OR "Research programmes".ti OR "Research programme".ti OR "Research methods".ti OR "Research method".ti OR "Research questions".ti OR "Research question".ti OR "Participatory research".ti OR "co research*".ti OR coresearch*.ti OR "Behavioral Research".ti OR "Health Services Research".ti OR "Community-Based Participatory Research".ti OR "Comparative Effectiveness Research".ti OR "Global Burden of Disease".ti OR "Health Care Survey".ti OR "Health Care Surveys".ti OR "Health Impact Assessment".ti OR "Health Services Needs and Demand".ti OR "Needs Assessment".ti OR "Organizational Case Studies".ti OR "Organizational Case Study".ti OR "Patient Reported Outcome Measure".ti OR "Patient Reported Outcome Measures".ti OR "Human Experimentation".ti OR "Nursing Research".ti OR "Outcome Assessment".ti OR "Treatment Outcome".ti OR "Pharmaceutical Research".ti OR "Pharmacy Research".ti OR "Rehabilitation Research".ti OR "Community-Based Participatory Research".ti OR *"Participatory Research"/ OR *"Empirical Research"/ OR exp *"Grounded Theory"/ OR exp *"Qualitative Research"/ OR exp *"Interdisciplinary Research"/ OR exp *"Operations Research"/ OR exp *"Public Health Systems Research"/ OR ***"Methodology"**/ OR "Empirical Research".ti OR "Grounded Theory".ti OR "Qualitative Research".ti OR "Interdisciplinary Research".ti OR "Operations Research".ti OR "Peer Review, Research".ti OR "Public Health Systems Research".ti OR "Research Design".ti OR "Research Report".ti OR *"Research Subject"/ OR "Research Subjects".ti OR "Research Subject".ti OR "Participatory Research".ti **OR "research".ti**) AND (Reform*.mp OR Develop*.mp OR Change*.mp OR Design*.mp OR Redesign*.mp OR "Conceptualization".mp OR "Conceptualisation".mp OR concept*.mp OR Model*.mp OR Method*.mp OR "strategy".mp OR "strategies".mp OR strateg*.mp))

1. **Web of Science**

(ts=("patient participation" OR "patient participation" OR "client participation" OR "consumer participation" OR "user participation" OR "carer participation" OR "caregiver participation" OR "public participation" OR "citizen participation" OR "stakeholder participation" OR "representative participation" OR "relative participation" OR "family participation" OR "lay participation" OR "resident participation" OR "patient involvement" OR "client involvement" OR "consumer involvement" OR "user involvement" OR "carer involvement" OR "caregiver involvement" OR "public involvement" OR "citizen involvement" OR "stakeholder involvement" OR "representative involvement" OR "relative involvement" OR "family involvement" OR "lay involvement" OR "resident involvement" OR "patient engagement" OR "client engagement" OR "consumer engagement" OR "user engagement" OR "carer engagement" OR "caregiver engagement" OR "public engagement" OR "citizen engagement" OR "stakeholder engagement" OR "representative engagement" OR "relative engagement" OR "family engagement" OR "lay engagement" OR "resident engagement" OR "patient collaboration" OR "client collaboration" OR "consumer collaboration" OR "user collaboration" OR "carer collaboration" OR "caregiver collaboration" OR "public collaboration" OR "citizen collaboration" OR "stakeholder collaboration" OR "representative collaboration" OR "relative collaboration" OR "family collaboration" OR "lay collaboration" OR "resident collaboration" OR "patient partnership" OR "client partnership" OR "consumer partnership" OR "user partnership" OR "carer partnership" OR "caregiver partnership" OR "public partnership" OR "citizen partnership" OR "stakeholder partnership" OR "representative partnership" OR "relative partnership" OR "family partnership" OR "lay partnership" OR "resident partnership" OR "patient empowerment" OR "client empowerment" OR "consumer empowerment" OR "user empowerment" OR "carer empowerment" OR "caregiver empowerment" OR "public empowerment" OR "citizen empowerment" OR "stakeholder empowerment" OR "representative empowerment" OR "relative empowerment" OR "family empowerment" OR "lay empowerment" OR "resident empowerment" OR "patient consultation" OR "client consultation" OR "consumer consultation" OR "user consultation" OR "carer consultation" OR "caregiver consultation" OR "public consultation" OR "citizen consultation" OR "stakeholder consultation" OR "representative consultation" OR "relative consultation" OR "family consultation" OR "lay consultation" OR "resident consultation" OR "Patient Activation" OR "Patient Activations" OR "co-research*" OR coresearch*) AND ts=("Residential Home" OR "Residential Facilities" OR "Residential Facility" OR "Nursing Home" OR "Nursing Homes" OR "Nursing Home" OR "Institutionalization" OR "institutional care" OR "Institutionalization" OR "Institutionalisation" OR "Long Term Care" OR "Long-Term Care" OR "nursing home patient" OR "Home resident" OR "Home residents" OR exp "Dementia" OR "dementia" OR dement* OR alzheimer* OR exp "Mental health care" OR "Mental healthcare" OR "Mental health care" OR exp "Mental Health Service" OR "Mental Health Services" OR "Mental Health Service" OR exp "Disabled Person" OR "Disabled" OR "Cognitive impair*" OR exp "Cognitive Defect") AND ti=("Research" OR "Medical research" OR "Medical research" OR "Biomedical research" OR "Care research" OR "Healthcare research" OR "Scientific research" OR "Research agenda" OR "Research agendas" OR "research priorities" OR "research priority" OR "Research programs" OR "Research program" OR "Research programmes" OR "Research programme" OR "Research methods" OR "Research method" OR "Research questions" OR "Research question" OR "Participatory research" OR "co research*" OR coresearch* OR "Behavioral Research" OR "Health Services Research" OR "Community-Based Participatory Research" OR "Comparative Effectiveness Research" OR "Global Burden of Disease" OR "Health Care Survey" OR "Health Care Surveys" OR "Health Impact Assessment" OR "Health Services Needs and Demand" OR "Needs Assessment" OR "Organizational Case Studies" OR "Organizational Case Study" OR "Patient Reported Outcome Measure" OR "Patient Reported Outcome Measures" OR "Human Experimentation" OR "Nursing Research" OR "Outcome Assessment" OR "Treatment Outcome" OR "Pharmaceutical Research" OR "Pharmacy Research" OR "Rehabilitation Research" OR "Community-Based Participatory Research" OR "Participatory Research" OR "Empirical Research" OR "Grounded Theory" OR "Qualitative Research" OR "Interdisciplinary Research" OR "Operations Research" OR "Public Health Systems Research" OR **"Methodology"** OR "Empirical Research" OR "Grounded Theory" OR "Qualitative Research" OR "Interdisciplinary Research" OR "Operations Research" OR "Peer Review, Research" OR "Public Health Systems Research" OR "Research Design" OR "Research Report" OR "Research Subject" OR "Research Subjects" OR "Research Subject" OR "Participatory Research" **OR "research"**) AND ts=(Reform* OR Develop* OR Change* OR Design* OR Redesign* OR "Conceptualization" OR "Conceptualisation" OR concept* OR Model* OR Method* OR "strategy" OR "strategies" OR strateg*))

1. **Cochrane**

(("patient participation" OR "patient participation" OR "client participation" OR "consumer participation" OR "user participation" OR "carer participation" OR "caregiver participation" OR "public participation" OR "citizen participation" OR "stakeholder participation" OR "representative participation" OR "relative participation" OR "family participation" OR "lay participation" OR "resident participation" OR "patient involvement" OR "client involvement" OR "consumer involvement" OR "user involvement" OR "carer involvement" OR "caregiver involvement" OR "public involvement" OR "citizen involvement" OR "stakeholder involvement" OR "representative involvement" OR "relative involvement" OR "family involvement" OR "lay involvement" OR "resident involvement" OR "patient engagement" OR "client engagement" OR "consumer engagement" OR "user engagement" OR "carer engagement" OR "caregiver engagement" OR "public engagement" OR "citizen engagement" OR "stakeholder engagement" OR "representative engagement" OR "relative engagement" OR "family engagement" OR "lay engagement" OR "resident engagement" OR "patient collaboration" OR "client collaboration" OR "consumer collaboration" OR "user collaboration" OR "carer collaboration" OR "caregiver collaboration" OR "public collaboration" OR "citizen collaboration" OR "stakeholder collaboration" OR "representative collaboration" OR "relative collaboration" OR "family collaboration" OR "lay collaboration" OR "resident collaboration" OR "patient partnership" OR "client partnership" OR "consumer partnership" OR "user partnership" OR "carer partnership" OR "caregiver partnership" OR "public partnership" OR "citizen partnership" OR "stakeholder partnership" OR "representative partnership" OR "relative partnership" OR "family partnership" OR "lay partnership" OR "resident partnership" OR "patient empowerment" OR "client empowerment" OR "consumer empowerment" OR "user empowerment" OR "carer empowerment" OR "caregiver empowerment" OR "public empowerment" OR "citizen empowerment" OR "stakeholder empowerment" OR "representative empowerment" OR "relative empowerment" OR "family empowerment" OR "lay empowerment" OR "resident empowerment" OR "patient consultation" OR "client consultation" OR "consumer consultation" OR "user consultation" OR "carer consultation" OR "caregiver consultation" OR "public consultation" OR "citizen consultation" OR "stakeholder consultation" OR "representative consultation" OR "relative consultation" OR "family consultation" OR "lay consultation" OR "resident consultation" OR "Patient Activation" OR "Patient Activations" OR "co-research*" OR coresearch*):ti,ab,kw AND ("Residential Home" OR "Residential Facilities" OR "Residential Facility" OR "Nursing Home" OR "Nursing Homes" OR "Nursing Home" OR "Institutionalization" OR "institutional care" OR "Institutionalization" OR "Institutionalisation" OR "Long Term Care" OR "Long-Term Care" OR "nursing home patient" OR "Home resident" OR "Home residents" OR exp "Dementia" OR "dementia" OR dement* OR alzheimer* OR exp "Mental health care" OR "Mental healthcare" OR "Mental health care" OR exp "Mental Health Service" OR "Mental Health Services" OR "Mental Health Service" OR exp "Disabled Person" OR "Disabled" OR "Cognitive impair*" OR exp "Cognitive Defect"):ti,ab,kw AND ("Research" OR "Medical research" OR "Medical research" OR "Biomedical research" OR "Care research" OR "Healthcare research" OR "Scientific research" OR "Research agenda" OR "Research agendas" OR "research priorities" OR "research priority" OR "Research programs" OR "Research program" OR "Research programmes" OR "Research programme" OR "Research methods" OR "Research method" OR "Research questions" OR "Research question" OR "Participatory research" OR "co research*" OR coresearch* OR "Behavioral Research" OR "Health Services Research" OR "Community-Based Participatory Research" OR "Comparative Effectiveness Research" OR "Global Burden of Disease" OR "Health Care Survey" OR "Health Care Surveys" OR "Health Impact Assessment" OR "Health Services Needs and Demand" OR "Needs Assessment" OR "Organizational Case Studies" OR "Organizational Case Study" OR "Patient Reported Outcome Measure" OR "Patient Reported Outcome Measures" OR "Human Experimentation" OR "Nursing Research" OR "Outcome Assessment" OR "Treatment Outcome" OR "Pharmaceutical Research" OR "Pharmacy Research" OR "Rehabilitation Research" OR "Community-Based Participatory Research" OR "Participatory Research" OR "Empirical Research" OR "Grounded Theory" OR "Qualitative Research" OR "Interdisciplinary Research" OR "Operations Research" OR "Public Health Systems Research" OR **"Methodology"** OR "Empirical Research" OR "Grounded Theory" OR "Qualitative Research" OR "Interdisciplinary Research" OR "Operations Research" OR "Peer Review, Research" OR "Public Health Systems Research" OR "Research Design" OR "Research Report" OR "Research Subject" OR "Research Subjects" OR "Research Subject" OR "Participatory Research" **OR "research"**):ti AND (Reform* OR Develop* OR Change* OR Design* OR Redesign* OR "Conceptualization" OR "Conceptualisation" OR concept* OR Model* OR Method* OR "strategy" OR "strategies" OR strateg*):ti,ab,kw)

1. **PsycINFO**

(TI(("patient participation" OR "patient participation" OR "client participation" OR "consumer participation" OR "user participation" OR "carer participation" OR "caregiver participation" OR "public participation" OR "citizen participation" OR "stakeholder participation" OR "representative participation" OR "relative participation" OR "family participation" OR "lay participation" OR "resident participation" OR "patient involvement" OR "client involvement" OR "consumer involvement" OR "user involvement" OR "carer involvement" OR "caregiver involvement" OR "public involvement" OR "citizen involvement" OR "stakeholder involvement" OR "representative involvement" OR "relative involvement" OR "family involvement" OR "lay involvement" OR "resident involvement" OR "patient engagement" OR "client engagement" OR "consumer engagement" OR "user engagement" OR "carer engagement" OR "caregiver engagement" OR "public engagement" OR "citizen engagement" OR "stakeholder engagement" OR "representative engagement" OR "relative engagement" OR "family engagement" OR "lay engagement" OR "resident engagement" OR "patient collaboration" OR "client collaboration" OR "consumer collaboration" OR "user collaboration" OR "carer collaboration" OR "caregiver collaboration" OR "public collaboration" OR "citizen collaboration" OR "stakeholder collaboration" OR "representative collaboration" OR "relative collaboration" OR "family collaboration" OR "lay collaboration" OR "resident collaboration" OR "patient partnership" OR "client partnership" OR "consumer partnership" OR "user partnership" OR "carer partnership" OR "caregiver partnership" OR "public partnership" OR "citizen partnership" OR "stakeholder partnership" OR "representative partnership" OR "relative partnership" OR "family partnership" OR "lay partnership" OR "resident partnership" OR "patient empowerment" OR "client empowerment" OR "consumer empowerment" OR "user empowerment" OR "carer empowerment" OR "caregiver empowerment" OR "public empowerment" OR "citizen empowerment" OR "stakeholder empowerment" OR "representative empowerment" OR "relative empowerment" OR "family empowerment" OR "lay empowerment" OR "resident empowerment" OR "patient consultation" OR "client consultation" OR "consumer consultation" OR "user consultation" OR "carer consultation" OR "caregiver consultation" OR "public consultation" OR "citizen consultation" OR "stakeholder consultation" OR "representative consultation" OR "relative consultation" OR "family consultation" OR "lay consultation" OR "resident consultation" OR "Patient Activation" OR "Patient Activations" OR "co-research*" OR coresearch*) AND ("Residential Home" OR "Residential Facilities" OR "Residential Facility" OR "Nursing Home" OR "Nursing Homes" OR "Nursing Home" OR "Institutionalization" OR "institutional care" OR "Institutionalization" OR "Institutionalisation" OR "Long Term Care" OR "Long-Term Care" OR "nursing home patient" OR "Home resident" OR "Home residents" OR exp "Dementia" OR "dementia" OR dement* OR alzheimer* OR exp "Mental health care" OR "Mental healthcare" OR "Mental health care" OR exp "Mental Health Service" OR "Mental Health Services" OR "Mental Health Service" OR exp "Disabled Person" OR "Disabled" OR "Cognitive impair*" OR exp "Cognitive Defect") AND (Reform* OR Develop* OR Change* OR Design* OR Redesign* OR "Conceptualization" OR "Conceptualisation" OR concept* OR Model* OR Method* OR "strategy" OR "strategies" OR strateg*)) AND TI("Research" OR "Medical research" OR "Medical research" OR "Biomedical research" OR "Care research" OR "Healthcare research" OR "Scientific research" OR "Research agenda" OR "Research agendas" OR "research priorities" OR "research priority" OR "Research programs" OR "Research program" OR "Research programmes" OR "Research programme" OR "Research methods" OR "Research method" OR "Research questions" OR "Research question" OR "Participatory research" OR "co research*" OR coresearch* OR "Behavioral Research" OR "Health Services Research" OR "Community-Based Participatory Research" OR "Comparative Effectiveness Research" OR "Global Burden of Disease" OR "Health Care Survey" OR "Health Care Surveys" OR "Health Impact Assessment" OR "Health Services Needs and Demand" OR "Needs Assessment" OR "Organizational Case Studies" OR "Organizational Case Study" OR "Patient Reported Outcome Measure" OR "Patient Reported Outcome Measures" OR "Human Experimentation" OR "Nursing Research" OR "Outcome Assessment" OR "Treatment Outcome" OR "Pharmaceutical Research" OR "Pharmacy Research" OR "Rehabilitation Research" OR "Community-Based Participatory Research" OR "Participatory Research" OR "Empirical Research" OR "Grounded Theory" OR "Qualitative Research" OR "Interdisciplinary Research" OR "Operations Research" OR "Public Health Systems Research" OR **"Methodology"** OR "Empirical Research" OR "Grounded Theory" OR "Qualitative Research" OR "Interdisciplinary Research" OR "Operations Research" OR "Peer Review, Research" OR "Public Health Systems Research" OR "Research Design" OR "Research Report" OR "Research Subject" OR "Research Subjects" OR "Research Subject" OR "Participatory Research" **OR "research"**)) OR (SU(("patient participation" OR "patient participation" OR "client participation" OR "consumer participation" OR "user participation" OR "carer participation" OR "caregiver participation" OR "public participation" OR "citizen participation" OR "stakeholder participation" OR "representative participation" OR "relative participation" OR "family participation" OR "lay participation" OR "resident participation" OR "patient involvement" OR "client involvement" OR "consumer involvement" OR "user involvement" OR "carer involvement" OR "caregiver involvement" OR "public involvement" OR "citizen involvement" OR "stakeholder involvement" OR "representative involvement" OR "relative involvement" OR "family involvement" OR "lay involvement" OR "resident involvement" OR "patient engagement" OR "client engagement" OR "consumer engagement" OR "user engagement" OR "carer engagement" OR "caregiver engagement" OR "public engagement" OR "citizen engagement" OR "stakeholder engagement" OR "representative engagement" OR "relative engagement" OR "family engagement" OR "lay engagement" OR "resident engagement" OR "patient collaboration" OR "client collaboration" OR "consumer collaboration" OR "user collaboration" OR "carer collaboration" OR "caregiver collaboration" OR "public collaboration" OR "citizen collaboration" OR "stakeholder collaboration" OR "representative collaboration" OR "relative collaboration" OR "family collaboration" OR "lay collaboration" OR "resident collaboration" OR "patient partnership" OR "client partnership" OR "consumer partnership" OR "user partnership" OR "carer partnership" OR "caregiver partnership" OR "public partnership" OR "citizen partnership" OR "stakeholder partnership" OR "representative partnership" OR "relative partnership" OR "family partnership" OR "lay partnership" OR "resident partnership" OR "patient empowerment" OR "client empowerment" OR "consumer empowerment" OR "user empowerment" OR "carer empowerment" OR "caregiver empowerment" OR "public empowerment" OR "citizen empowerment" OR "stakeholder empowerment" OR "representative empowerment" OR "relative empowerment" OR "family empowerment" OR "lay empowerment" OR "resident empowerment" OR "patient consultation" OR "client consultation" OR "consumer consultation" OR "user consultation" OR "carer consultation" OR "caregiver consultation" OR "public consultation" OR "citizen consultation" OR "stakeholder consultation" OR "representative consultation" OR "relative consultation" OR "family consultation" OR "lay consultation" OR "resident consultation" OR "Patient Activation" OR "Patient Activations" OR "co-research*" OR coresearch*) AND ("Residential Home" OR "Residential Facilities" OR "Residential Facility" OR "Nursing Home" OR "Nursing Homes" OR "Nursing Home" OR "Institutionalization" OR "institutional care" OR "Institutionalization" OR "Institutionalisation" OR "Long Term Care" OR "Long-Term Care" OR "nursing home patient" OR "Home resident" OR "Home residents" OR exp "Dementia" OR "dementia" OR dement* OR alzheimer* OR exp "Mental health care" OR "Mental healthcare" OR "Mental health care" OR exp "Mental Health Service" OR "Mental Health Services" OR "Mental Health Service" OR exp "Disabled Person" OR "Disabled" OR "Cognitive impair*" OR exp "Cognitive Defect") AND (Reform* OR Develop* OR Change* OR Design* OR Redesign* OR "Conceptualization" OR "Conceptualisation" OR concept* OR Model* OR Method* OR "strategy" OR "strategies" OR strateg*)) AND TI("Research" OR "Medical research" OR "Medical research" OR "Biomedical research" OR "Care research" OR "Healthcare research" OR "Scientific research" OR "Research agenda" OR "Research agendas" OR "research priorities" OR "research priority" OR "Research programs" OR "Research program" OR "Research programmes" OR "Research programme" OR "Research methods" OR "Research method" OR "Research questions" OR "Research question" OR "Participatory research" OR "co research*" OR coresearch* OR "Behavioral Research" OR "Health Services Research" OR "Community-Based Participatory Research" OR "Comparative Effectiveness Research" OR "Global Burden of Disease" OR "Health Care Survey" OR "Health Care Surveys" OR "Health Impact Assessment" OR "Health Services Needs and Demand" OR "Needs Assessment" OR "Organizational Case Studies" OR "Organizational Case Study" OR "Patient Reported Outcome Measure" OR "Patient Reported Outcome Measures" OR "Human Experimentation" OR "Nursing Research" OR "Outcome Assessment" OR "Treatment Outcome" OR "Pharmaceutical Research" OR "Pharmacy Research" OR "Rehabilitation Research" OR "Community-Based Participatory Research" OR "Participatory Research" OR "Empirical Research" OR "Grounded Theory" OR "Qualitative Research" OR "Interdisciplinary Research" OR "Operations Research" OR "Public Health Systems Research" OR **"Methodology"** OR "Empirical Research" OR "Grounded Theory" OR "Qualitative Research" OR "Interdisciplinary Research" OR "Operations Research" OR "Peer Review, Research" OR "Public Health Systems Research" OR "Research Design" OR "Research Report" OR "Research Subject" OR "Research Subjects" OR "Research Subject" OR "Participatory Research" **OR "research"**)) OR (MA(("patient participation" OR "patient participation" OR "client participation" OR "consumer participation" OR "user participation" OR "carer participation" OR "caregiver participation" OR "public participation" OR "citizen participation" OR "stakeholder participation" OR "representative participation" OR "relative participation" OR "family participation" OR "lay participation" OR "resident participation" OR "patient involvement" OR "client involvement" OR "consumer involvement" OR "user involvement" OR "carer involvement" OR "caregiver involvement" OR "public involvement" OR "citizen involvement" OR "stakeholder involvement" OR "representative involvement" OR "relative involvement" OR "family involvement" OR "lay involvement" OR "resident involvement" OR "patient engagement" OR "client engagement" OR "consumer engagement" OR "user engagement" OR "carer engagement" OR "caregiver engagement" OR "public engagement" OR "citizen engagement" OR "stakeholder engagement" OR "representative engagement" OR "relative engagement" OR "family engagement" OR "lay engagement" OR "resident engagement" OR "patient collaboration" OR "client collaboration" OR "consumer collaboration" OR "user collaboration" OR "carer collaboration" OR "caregiver collaboration" OR "public collaboration" OR "citizen collaboration" OR "stakeholder collaboration" OR "representative collaboration" OR "relative collaboration" OR "family collaboration" OR "lay collaboration" OR "resident collaboration" OR "patient partnership" OR "client partnership" OR "consumer partnership" OR "user partnership" OR "carer partnership" OR "caregiver partnership" OR "public partnership" OR "citizen partnership" OR "stakeholder partnership" OR "representative partnership" OR "relative partnership" OR "family partnership" OR "lay partnership" OR "resident partnership" OR "patient empowerment" OR "client empowerment" OR "consumer empowerment" OR "user empowerment" OR "carer empowerment" OR "caregiver empowerment" OR "public empowerment" OR "citizen empowerment" OR "stakeholder empowerment" OR "representative empowerment" OR "relative empowerment" OR "family empowerment" OR "lay empowerment" OR "resident empowerment" OR "patient consultation" OR "client consultation" OR "consumer consultation" OR "user consultation" OR "carer consultation" OR "caregiver consultation" OR "public consultation" OR "citizen consultation" OR "stakeholder consultation" OR "representative consultation" OR "relative consultation" OR "family consultation" OR "lay consultation" OR "resident consultation" OR "Patient Activation" OR "Patient Activations" OR "co-research*" OR coresearch*) AND ("Residential Home" OR "Residential Facilities" OR "Residential Facility" OR "Nursing Home" OR "Nursing Homes" OR "Nursing Home" OR "Institutionalization" OR "institutional care" OR "Institutionalization" OR "Institutionalisation" OR "Long Term Care" OR "Long-Term Care" OR "nursing home patient" OR "Home resident" OR "Home residents" OR exp "Dementia" OR "dementia" OR dement* OR alzheimer* OR exp "Mental health care" OR "Mental healthcare" OR "Mental health care" OR exp "Mental Health Service" OR "Mental Health Services" OR "Mental Health Service" OR exp "Disabled Person" OR "Disabled" OR "Cognitive impair*" OR exp "Cognitive Defect") AND (Reform* OR Develop* OR Change* OR Design* OR Redesign* OR "Conceptualization" OR "Conceptualisation" OR concept* OR Model* OR Method* OR "strategy" OR "strategies" OR strateg*)) AND TI("Research" OR "Medical research" OR "Medical research" OR "Biomedical research" OR "Care research" OR "Healthcare research" OR "Scientific research" OR "Research agenda" OR "Research agendas" OR "research priorities" OR "research priority" OR "Research programs" OR "Research program" OR "Research programmes" OR "Research programme" OR "Research methods" OR "Research method" OR "Research questions" OR "Research question" OR "Participatory research" OR "co research*" OR coresearch* OR "Behavioral Research" OR "Health Services Research" OR "Community-Based Participatory Research" OR "Comparative Effectiveness Research" OR "Global Burden of Disease" OR "Health Care Survey" OR "Health Care Surveys" OR "Health Impact Assessment" OR "Health Services Needs and Demand" OR "Needs Assessment" OR "Organizational Case Studies" OR "Organizational Case Study" OR "Patient Reported Outcome Measure" OR "Patient Reported Outcome Measures" OR "Human Experimentation" OR "Nursing Research" OR "Outcome Assessment" OR "Treatment Outcome" OR "Pharmaceutical Research" OR "Pharmacy Research" OR "Rehabilitation Research" OR "Community-Based Participatory Research" OR "Participatory Research" OR "Empirical Research" OR "Grounded Theory" OR "Qualitative Research" OR "Interdisciplinary Research" OR "Operations Research" OR "Public Health Systems Research" OR **"Methodology"** OR "Empirical Research" OR "Grounded Theory" OR "Qualitative Research" OR "Interdisciplinary Research" OR "Operations Research" OR "Peer Review, Research" OR "Public Health Systems Research" OR "Research Design" OR "Research Report" OR "Research Subject" OR "Research Subjects" OR "Research Subject" OR "Participatory Research" **OR "research"**)) OR (AB(("patient participation" OR "patient participation" OR "client participation" OR "consumer participation" OR "user participation" OR "carer participation" OR "caregiver participation" OR "public participation" OR "citizen participation" OR "stakeholder participation" OR "representative participation" OR "relative participation" OR "family participation" OR "lay participation" OR "resident participation" OR "patient involvement" OR "client involvement" OR "consumer involvement" OR "user involvement" OR "carer involvement" OR "caregiver involvement" OR "public involvement" OR "citizen involvement" OR "stakeholder involvement" OR "representative involvement" OR "relative involvement" OR "family involvement" OR "lay involvement" OR "resident involvement" OR "patient engagement" OR "client engagement" OR "consumer engagement" OR "user engagement" OR "carer engagement" OR "caregiver engagement" OR "public engagement" OR "citizen engagement" OR "stakeholder engagement" OR "representative engagement" OR "relative engagement" OR "family engagement" OR "lay engagement" OR "resident engagement" OR "patient collaboration" OR "client collaboration" OR "consumer collaboration" OR "user collaboration" OR "carer collaboration" OR "caregiver collaboration" OR "public collaboration" OR "citizen collaboration" OR "stakeholder collaboration" OR "representative collaboration" OR "relative collaboration" OR "family collaboration" OR "lay collaboration" OR "resident collaboration" OR "patient partnership" OR "client partnership" OR "consumer partnership" OR "user partnership" OR "carer partnership" OR "caregiver partnership" OR "public partnership" OR "citizen partnership" OR "stakeholder partnership" OR "representative partnership" OR "relative partnership" OR "family partnership" OR "lay partnership" OR "resident partnership" OR "patient empowerment" OR "client empowerment" OR "consumer empowerment" OR "user empowerment" OR "carer empowerment" OR "caregiver empowerment" OR "public empowerment" OR "citizen empowerment" OR "stakeholder empowerment" OR "representative empowerment" OR "relative empowerment" OR "family empowerment" OR "lay empowerment" OR "resident empowerment" OR "patient consultation" OR "client consultation" OR "consumer consultation" OR "user consultation" OR "carer consultation" OR "caregiver consultation" OR "public consultation" OR "citizen consultation" OR "stakeholder consultation" OR "representative consultation" OR "relative consultation" OR "family consultation" OR "lay consultation" OR "resident consultation" OR "Patient Activation" OR "Patient Activations" OR "co-research*" OR coresearch*) AND ("Residential Home" OR "Residential Facilities" OR "Residential Facility" OR "Nursing Home" OR "Nursing Homes" OR "Nursing Home" OR "Institutionalization" OR "institutional care" OR "Institutionalization" OR "Institutionalisation" OR "Long Term Care" OR "Long-Term Care" OR "nursing home patient" OR "Home resident" OR "Home residents" OR exp "Dementia" OR "dementia" OR dement* OR alzheimer* OR exp "Mental health care" OR "Mental healthcare" OR "Mental health care" OR exp "Mental Health Service" OR "Mental Health Services" OR "Mental Health Service" OR exp "Disabled Person" OR "Disabled" OR "Cognitive impair*" OR exp "Cognitive Defect") AND (Reform* OR Develop* OR Change* OR Design* OR Redesign* OR "Conceptualization" OR "Conceptualisation" OR concept* OR Model* OR Method* OR "strategy" OR "strategies" OR strateg*)) AND TI("Research" OR "Medical research" OR "Medical research" OR "Biomedical research" OR "Care research" OR "Healthcare research" OR "Scientific research" OR "Research agenda" OR "Research agendas" OR "research priorities" OR "research priority" OR "Research programs" OR "Research program" OR "Research programmes" OR "Research programme" OR "Research methods" OR "Research method" OR "Research questions" OR "Research question" OR "Participatory research" OR "co research*" OR coresearch* OR "Behavioral Research" OR "Health Services Research" OR "Community-Based Participatory Research" OR "Comparative Effectiveness Research" OR "Global Burden of Disease" OR "Health Care Survey" OR "Health Care Surveys" OR "Health Impact Assessment" OR "Health Services Needs and Demand" OR "Needs Assessment" OR "Organizational Case Studies" OR "Organizational Case Study" OR "Patient Reported Outcome Measure" OR "Patient Reported Outcome Measures" OR "Human Experimentation" OR "Nursing Research" OR "Outcome Assessment" OR "Treatment Outcome" OR "Pharmaceutical Research" OR "Pharmacy Research" OR "Rehabilitation Research" OR "Community-Based Participatory Research" OR "Participatory Research" OR "Empirical Research" OR "Grounded Theory" OR "Qualitative Research" OR "Interdisciplinary Research" OR "Operations Research" OR "Public Health Systems Research" OR **"Methodology"** OR "Empirical Research" OR "Grounded Theory" OR "Qualitative Research" OR "Interdisciplinary Research" OR "Operations Research" OR "Peer Review, Research" OR "Public Health Systems Research" OR "Research Design" OR "Research Report" OR "Research Subject" OR "Research Subjects" OR "Research Subject" OR "Participatory Research" **OR "research"**))

1. **Academic Search Premier**

(TI(("patient participation" OR "patient participation" OR "client participation" OR "consumer participation" OR "user participation" OR "carer participation" OR "caregiver participation" OR "public participation" OR "citizen participation" OR "stakeholder participation" OR "representative participation" OR "relative participation" OR "family participation" OR "lay participation" OR "resident participation" OR "patient involvement" OR "client involvement" OR "consumer involvement" OR "user involvement" OR "carer involvement" OR "caregiver involvement" OR "public involvement" OR "citizen involvement" OR "stakeholder involvement" OR "representative involvement" OR "relative involvement" OR "family involvement" OR "lay involvement" OR "resident involvement" OR "patient engagement" OR "client engagement" OR "consumer engagement" OR "user engagement" OR "carer engagement" OR "caregiver engagement" OR "public engagement" OR "citizen engagement" OR "stakeholder engagement" OR "representative engagement" OR "relative engagement" OR "family engagement" OR "lay engagement" OR "resident engagement" OR "patient collaboration" OR "client collaboration" OR "consumer collaboration" OR "user collaboration" OR "carer collaboration" OR "caregiver collaboration" OR "public collaboration" OR "citizen collaboration" OR "stakeholder collaboration" OR "representative collaboration" OR "relative collaboration" OR "family collaboration" OR "lay collaboration" OR "resident collaboration" OR "patient partnership" OR "client partnership" OR "consumer partnership" OR "user partnership" OR "carer partnership" OR "caregiver partnership" OR "public partnership" OR "citizen partnership" OR "stakeholder partnership" OR "representative partnership" OR "relative partnership" OR "family partnership" OR "lay partnership" OR "resident partnership" OR "patient empowerment" OR "client empowerment" OR "consumer empowerment" OR "user empowerment" OR "carer empowerment" OR "caregiver empowerment" OR "public empowerment" OR "citizen empowerment" OR "stakeholder empowerment" OR "representative empowerment" OR "relative empowerment" OR "family empowerment" OR "lay empowerment" OR "resident empowerment" OR "patient consultation" OR "client consultation" OR "consumer consultation" OR "user consultation" OR "carer consultation" OR "caregiver consultation" OR "public consultation" OR "citizen consultation" OR "stakeholder consultation" OR "representative consultation" OR "relative consultation" OR "family consultation" OR "lay consultation" OR "resident consultation" OR "Patient Activation" OR "Patient Activations" OR "co-research*" OR coresearch*) AND ("Residential Home" OR "Residential Facilities" OR "Residential Facility" OR "Nursing Home" OR "Nursing Homes" OR "Nursing Home" OR "Institutionalization" OR "institutional care" OR "Institutionalization" OR "Institutionalisation" OR "Long Term Care" OR "Long-Term Care" OR "nursing home patient" OR "Home resident" OR "Home residents" OR exp "Dementia" OR "dementia" OR dement* OR alzheimer* OR exp "Mental health care" OR "Mental healthcare" OR "Mental health care" OR exp "Mental Health Service" OR "Mental Health Services" OR "Mental Health Service" OR exp "Disabled Person" OR "Disabled" OR "Cognitive impair*" OR exp "Cognitive Defect") AND (Reform* OR Develop* OR Change* OR Design* OR Redesign* OR "Conceptualization" OR "Conceptualisation" OR concept* OR Model* OR Method* OR "strategy" OR "strategies" OR strateg*)) AND TI("Research" OR "Medical research" OR "Medical research" OR "Biomedical research" OR "Care research" OR "Healthcare research" OR "Scientific research" OR "Research agenda" OR "Research agendas" OR "research priorities" OR "research priority" OR "Research programs" OR "Research program" OR "Research programmes" OR "Research programme" OR "Research methods" OR "Research method" OR "Research questions" OR "Research question" OR "Participatory research" OR "co research*" OR coresearch* OR "Behavioral Research" OR "Health Services Research" OR "Community-Based Participatory Research" OR "Comparative Effectiveness Research" OR "Global Burden of Disease" OR "Health Care Survey" OR "Health Care Surveys" OR "Health Impact Assessment" OR "Health Services Needs and Demand" OR "Needs Assessment" OR "Organizational Case Studies" OR "Organizational Case Study" OR "Patient Reported Outcome Measure" OR "Patient Reported Outcome Measures" OR "Human Experimentation" OR "Nursing Research" OR "Outcome Assessment" OR "Treatment Outcome" OR "Pharmaceutical Research" OR "Pharmacy Research" OR "Rehabilitation Research" OR "Community-Based Participatory Research" OR "Participatory Research" OR "Empirical Research" OR "Grounded Theory" OR "Qualitative Research" OR "Interdisciplinary Research" OR "Operations Research" OR "Public Health Systems Research" OR **"Methodology"** OR "Empirical Research" OR "Grounded Theory" OR "Qualitative Research" OR "Interdisciplinary Research" OR "Operations Research" OR "Peer Review, Research" OR "Public Health Systems Research" OR "Research Design" OR "Research Report" OR "Research Subject" OR "Research Subjects" OR "Research Subject" OR "Participatory Research" **OR "research"**)) OR (SU(("patient participation" OR "patient participation" OR "client participation" OR "consumer participation" OR "user participation" OR "carer participation" OR "caregiver participation" OR "public participation" OR "citizen participation" OR "stakeholder participation" OR "representative participation" OR "relative participation" OR "family participation" OR "lay participation" OR "resident participation" OR "patient involvement" OR "client involvement" OR "consumer involvement" OR "user involvement" OR "carer involvement" OR "caregiver involvement" OR "public involvement" OR "citizen involvement" OR "stakeholder involvement" OR "representative involvement" OR "relative involvement" OR "family involvement" OR "lay involvement" OR "resident involvement" OR "patient engagement" OR "client engagement" OR "consumer engagement" OR "user engagement" OR "carer engagement" OR "caregiver engagement" OR "public engagement" OR "citizen engagement" OR "stakeholder engagement" OR "representative engagement" OR "relative engagement" OR "family engagement" OR "lay engagement" OR "resident engagement" OR "patient collaboration" OR "client collaboration" OR "consumer collaboration" OR "user collaboration" OR "carer collaboration" OR "caregiver collaboration" OR "public collaboration" OR "citizen collaboration" OR "stakeholder collaboration" OR "representative collaboration" OR "relative collaboration" OR "family collaboration" OR "lay collaboration" OR "resident collaboration" OR "patient partnership" OR "client partnership" OR "consumer partnership" OR "user partnership" OR "carer partnership" OR "caregiver partnership" OR "public partnership" OR "citizen partnership" OR "stakeholder partnership" OR "representative partnership" OR "relative partnership" OR "family partnership" OR "lay partnership" OR "resident partnership" OR "patient empowerment" OR "client empowerment" OR "consumer empowerment" OR "user empowerment" OR "carer empowerment" OR "caregiver empowerment" OR "public empowerment" OR "citizen empowerment" OR "stakeholder empowerment" OR "representative empowerment" OR "relative empowerment" OR "family empowerment" OR "lay empowerment" OR "resident empowerment" OR "patient consultation" OR "client consultation" OR "consumer consultation" OR "user consultation" OR "carer consultation" OR "caregiver consultation" OR "public consultation" OR "citizen consultation" OR "stakeholder consultation" OR "representative consultation" OR "relative consultation" OR "family consultation" OR "lay consultation" OR "resident consultation" OR "Patient Activation" OR "Patient Activations" OR "co-research*" OR coresearch*) AND ("Residential Home" OR "Residential Facilities" OR "Residential Facility" OR "Nursing Home" OR "Nursing Homes" OR "Nursing Home" OR "Institutionalization" OR "institutional care" OR "Institutionalization" OR "Institutionalisation" OR "Long Term Care" OR "Long-Term Care" OR "nursing home patient" OR "Home resident" OR "Home residents" OR exp "Dementia" OR "dementia" OR dement* OR alzheimer* OR exp "Mental health care" OR "Mental healthcare" OR "Mental health care" OR exp "Mental Health Service" OR "Mental Health Services" OR "Mental Health Service" OR exp "Disabled Person" OR "Disabled" OR "Cognitive impair*" OR exp "Cognitive Defect") AND (Reform* OR Develop* OR Change* OR Design* OR Redesign* OR "Conceptualization" OR "Conceptualisation" OR concept* OR Model* OR Method* OR "strategy" OR "strategies" OR strateg*)) AND TI("Research" OR "Medical research" OR "Medical research" OR "Biomedical research" OR "Care research" OR "Healthcare research" OR "Scientific research" OR "Research agenda" OR "Research agendas" OR "research priorities" OR "research priority" OR "Research programs" OR "Research program" OR "Research programmes" OR "Research programme" OR "Research methods" OR "Research method" OR "Research questions" OR "Research question" OR "Participatory research" OR "co research*" OR coresearch* OR "Behavioral Research" OR "Health Services Research" OR "Community-Based Participatory Research" OR "Comparative Effectiveness Research" OR "Global Burden of Disease" OR "Health Care Survey" OR "Health Care Surveys" OR "Health Impact Assessment" OR "Health Services Needs and Demand" OR "Needs Assessment" OR "Organizational Case Studies" OR "Organizational Case Study" OR "Patient Reported Outcome Measure" OR "Patient Reported Outcome Measures" OR "Human Experimentation" OR "Nursing Research" OR "Outcome Assessment" OR "Treatment Outcome" OR "Pharmaceutical Research" OR "Pharmacy Research" OR "Rehabilitation Research" OR "Community-Based Participatory Research" OR "Participatory Research" OR "Empirical Research" OR "Grounded Theory" OR "Qualitative Research" OR "Interdisciplinary Research" OR "Operations Research" OR "Public Health Systems Research" OR **"Methodology"** OR "Empirical Research" OR "Grounded Theory" OR "Qualitative Research" OR "Interdisciplinary Research" OR "Operations Research" OR "Peer Review, Research" OR "Public Health Systems Research" OR "Research Design" OR "Research Report" OR "Research Subject" OR "Research Subjects" OR "Research Subject" OR "Participatory Research" **OR "research"**)) OR (KW(("patient participation" OR "patient participation" OR "client participation" OR "consumer participation" OR "user participation" OR "carer participation" OR "caregiver participation" OR "public participation" OR "citizen participation" OR "stakeholder participation" OR "representative participation" OR "relative participation" OR "family participation" OR "lay participation" OR "resident participation" OR "patient involvement" OR "client involvement" OR "consumer involvement" OR "user involvement" OR "carer involvement" OR "caregiver involvement" OR "public involvement" OR "citizen involvement" OR "stakeholder involvement" OR "representative involvement" OR "relative involvement" OR "family involvement" OR "lay involvement" OR "resident involvement" OR "patient engagement" OR "client engagement" OR "consumer engagement" OR "user engagement" OR "carer engagement" OR "caregiver engagement" OR "public engagement" OR "citizen engagement" OR "stakeholder engagement" OR "representative engagement" OR "relative engagement" OR "family engagement" OR "lay engagement" OR "resident engagement" OR "patient collaboration" OR "client collaboration" OR "consumer collaboration" OR "user collaboration" OR "carer collaboration" OR "caregiver collaboration" OR "public collaboration" OR "citizen collaboration" OR "stakeholder collaboration" OR "representative collaboration" OR "relative collaboration" OR "family collaboration" OR "lay collaboration" OR "resident collaboration" OR "patient partnership" OR "client partnership" OR "consumer partnership" OR "user partnership" OR "carer partnership" OR "caregiver partnership" OR "public partnership" OR "citizen partnership" OR "stakeholder partnership" OR "representative partnership" OR "relative partnership" OR "family partnership" OR "lay partnership" OR "resident partnership" OR "patient empowerment" OR "client empowerment" OR "consumer empowerment" OR "user empowerment" OR "carer empowerment" OR "caregiver empowerment" OR "public empowerment" OR "citizen empowerment" OR "stakeholder empowerment" OR "representative empowerment" OR "relative empowerment" OR "family empowerment" OR "lay empowerment" OR "resident empowerment" OR "patient consultation" OR "client consultation" OR "consumer consultation" OR "user consultation" OR "carer consultation" OR "caregiver consultation" OR "public consultation" OR "citizen consultation" OR "stakeholder consultation" OR "representative consultation" OR "relative consultation" OR "family consultation" OR "lay consultation" OR "resident consultation" OR "Patient Activation" OR "Patient Activations" OR "co-research*" OR coresearch*) AND ("Residential Home" OR "Residential Facilities" OR "Residential Facility" OR "Nursing Home" OR "Nursing Homes" OR "Nursing Home" OR "Institutionalization" OR "institutional care" OR "Institutionalization" OR "Institutionalisation" OR "Long Term Care" OR "Long-Term Care" OR "nursing home patient" OR "Home resident" OR "Home residents" OR exp "Dementia" OR "dementia" OR dement* OR alzheimer* OR exp "Mental health care" OR "Mental healthcare" OR "Mental health care" OR exp "Mental Health Service" OR "Mental Health Services" OR "Mental Health Service" OR exp "Disabled Person" OR "Disabled" OR "Cognitive impair*" OR exp "Cognitive Defect") AND (Reform* OR Develop* OR Change* OR Design* OR Redesign* OR "Conceptualization" OR "Conceptualisation" OR concept* OR Model* OR Method* OR "strategy" OR "strategies" OR strateg*)) AND TI("Research" OR "Medical research" OR "Medical research" OR "Biomedical research" OR "Care research" OR "Healthcare research" OR "Scientific research" OR "Research agenda" OR "Research agendas" OR "research priorities" OR "research priority" OR "Research programs" OR "Research program" OR "Research programmes" OR "Research programme" OR "Research methods" OR "Research method" OR "Research questions" OR "Research question" OR "Participatory research" OR "co research*" OR coresearch* OR "Behavioral Research" OR "Health Services Research" OR "Community-Based Participatory Research" OR "Comparative Effectiveness Research" OR "Global Burden of Disease" OR "Health Care Survey" OR "Health Care Surveys" OR "Health Impact Assessment" OR "Health Services Needs and Demand" OR "Needs Assessment" OR "Organizational Case Studies" OR "Organizational Case Study" OR "Patient Reported Outcome Measure" OR "Patient Reported Outcome Measures" OR "Human Experimentation" OR "Nursing Research" OR "Outcome Assessment" OR "Treatment Outcome" OR "Pharmaceutical Research" OR "Pharmacy Research" OR "Rehabilitation Research" OR "Community-Based Participatory Research" OR "Participatory Research" OR "Empirical Research" OR "Grounded Theory" OR "Qualitative Research" OR "Interdisciplinary Research" OR "Operations Research" OR "Public Health Systems Research" OR **"Methodology"** OR "Empirical Research" OR "Grounded Theory" OR "Qualitative Research" OR "Interdisciplinary Research" OR "Operations Research" OR "Peer Review, Research" OR "Public Health Systems Research" OR "Research Design" OR "Research Report" OR "Research Subject" OR "Research Subjects" OR "Research Subject" OR "Participatory Research" **OR "research"**)) OR (AB(("patient participation" OR "patient participation" OR "client participation" OR "consumer participation" OR "user participation" OR "carer participation" OR "caregiver participation" OR "public participation" OR "citizen participation" OR "stakeholder participation" OR "representative participation" OR "relative participation" OR "family participation" OR "lay participation" OR "resident participation" OR "patient involvement" OR "client involvement" OR "consumer involvement" OR "user involvement" OR "carer involvement" OR "caregiver involvement" OR "public involvement" OR "citizen involvement" OR "stakeholder involvement" OR "representative involvement" OR "relative involvement" OR "family involvement" OR "lay involvement" OR "resident involvement" OR "patient engagement" OR "client engagement" OR "consumer engagement" OR "user engagement" OR "carer engagement" OR "caregiver engagement" OR "public engagement" OR "citizen engagement" OR "stakeholder engagement" OR "representative engagement" OR "relative engagement" OR "family engagement" OR "lay engagement" OR "resident engagement" OR "patient collaboration" OR "client collaboration" OR "consumer collaboration" OR "user collaboration" OR "carer collaboration" OR "caregiver collaboration" OR "public collaboration" OR "citizen collaboration" OR "stakeholder collaboration" OR "representative collaboration" OR "relative collaboration" OR "family collaboration" OR "lay collaboration" OR "resident collaboration" OR "patient partnership" OR "client partnership" OR "consumer partnership" OR "user partnership" OR "carer partnership" OR "caregiver partnership" OR "public partnership" OR "citizen partnership" OR "stakeholder partnership" OR "representative partnership" OR "relative partnership" OR "family partnership" OR "lay partnership" OR "resident partnership" OR "patient empowerment" OR "client empowerment" OR "consumer empowerment" OR "user empowerment" OR "carer empowerment" OR "caregiver empowerment" OR "public empowerment" OR "citizen empowerment" OR "stakeholder empowerment" OR "representative empowerment" OR "relative empowerment" OR "family empowerment" OR "lay empowerment" OR "resident empowerment" OR "patient consultation" OR "client consultation" OR "consumer consultation" OR "user consultation" OR "carer consultation" OR "caregiver consultation" OR "public consultation" OR "citizen consultation" OR "stakeholder consultation" OR "representative consultation" OR "relative consultation" OR "family consultation" OR "lay consultation" OR "resident consultation" OR "Patient Activation" OR "Patient Activations" OR "co-research*" OR coresearch*) AND ("Residential Home" OR "Residential Facilities" OR "Residential Facility" OR "Nursing Home" OR "Nursing Homes" OR "Nursing Home" OR "Institutionalization" OR "institutional care" OR "Institutionalization" OR "Institutionalisation" OR "Long Term Care" OR "Long-Term Care" OR "nursing home patient" OR "Home resident" OR "Home residents" OR exp "Dementia" OR "dementia" OR dement* OR alzheimer* OR exp "Mental health care" OR "Mental healthcare" OR "Mental health care" OR exp "Mental Health Service" OR "Mental Health Services" OR "Mental Health Service" OR exp "Disabled Person" OR "Disabled" OR "Cognitive impair*" OR exp "Cognitive Defect") AND (Reform* OR Develop* OR Change* OR Design* OR Redesign* OR "Conceptualization" OR "Conceptualisation" OR concept* OR Model* OR Method* OR "strategy" OR "strategies" OR strateg*)) AND TI("Research" OR "Medical research" OR "Medical research" OR "Biomedical research" OR "Care research" OR "Healthcare research" OR "Scientific research" OR "Research agenda" OR "Research agendas" OR "research priorities" OR "research priority" OR "Research programs" OR "Research program" OR "Research programmes" OR "Research programme" OR "Research methods" OR "Research method" OR "Research questions" OR "Research question" OR "Participatory research" OR "co research*" OR coresearch* OR "Behavioral Research" OR "Health Services Research" OR "Community-Based Participatory Research" OR "Comparative Effectiveness Research" OR "Global Burden of Disease" OR "Health Care Survey" OR "Health Care Surveys" OR "Health Impact Assessment" OR "Health Services Needs and Demand" OR "Needs Assessment" OR "Organizational Case Studies" OR "Organizational Case Study" OR "Patient Reported Outcome Measure" OR "Patient Reported Outcome Measures" OR "Human Experimentation" OR "Nursing Research" OR "Outcome Assessment" OR "Treatment Outcome" OR "Pharmaceutical Research" OR "Pharmacy Research" OR "Rehabilitation Research" OR "Community-Based Participatory Research" OR "Participatory Research" OR "Empirical Research" OR "Grounded Theory" OR "Qualitative Research" OR "Interdisciplinary Research" OR "Operations Research" OR "Public Health Systems Research" OR **"Methodology"** OR "Empirical Research" OR "Grounded Theory" OR "Qualitative Research" OR "Interdisciplinary Research" OR "Operations Research" OR "Peer Review, Research" OR "Public Health Systems Research" OR "Research Design" OR "Research Report" OR "Research Subject" OR "Research Subjects" OR "Research Subject" OR "Participatory Research" **OR "research"**))

1. **JSTOR**

(ti:(participation OR involvement OR engagement OR collaboration OR partnership OR empowerment OR consultation OR Activation OR co-research OR coresearch) AND ti:(Nursing Home OR institutional care))

(ti:(participation OR involvement OR engagement OR collaboration OR partnership OR empowerment OR consultation OR Activation OR co-research OR coresearch) AND ti:(Long Term Care))

(ti:(participation OR involvement OR engagement OR collaboration OR partnership OR empowerment OR consultation OR Activation OR co-research OR coresearch) AND ti:(Dementia OR alzheimer))

(ti:(participation OR involvement OR engagement OR collaboration OR partnership OR empowerment OR consultation OR Activation OR co-research OR coresearch) AND ab:(Nursing Home OR institutional care))

(ti:(participation OR involvement OR engagement OR collaboration OR partnership OR empowerment OR consultation OR Activation OR co-research OR coresearch) AND ab:(Long Term Care))

(ti:(participation OR involvement OR engagement OR collaboration OR partnership OR empowerment OR consultation OR Activation OR co-research OR coresearch) AND ab:(Dementia OR alzheimer))

1. **Social Services Abstracts**

NOFT(("patient participation" OR "patient participation" OR "client participation" OR "consumer participation" OR "user participation" OR "carer participation" OR "caregiver participation" OR "public participation" OR "citizen participation" OR "stakeholder participation" OR "representative participation" OR "relative participation" OR "family participation" OR "lay participation" OR "resident participation" OR "patient involvement" OR "client involvement" OR "consumer involvement" OR "user involvement" OR "carer involvement" OR "caregiver involvement" OR "public involvement" OR "citizen involvement" OR "stakeholder involvement" OR "representative involvement" OR "relative involvement" OR "family involvement" OR "lay involvement" OR "resident involvement" OR "patient engagement" OR "client engagement" OR "consumer engagement" OR "user engagement" OR "carer engagement" OR "caregiver engagement" OR "public engagement" OR "citizen engagement" OR "stakeholder engagement" OR "representative engagement" OR "relative engagement" OR "family engagement" OR "lay engagement" OR "resident engagement" OR "patient collaboration" OR "client collaboration" OR "consumer collaboration" OR "user collaboration" OR "carer collaboration" OR "caregiver collaboration" OR "public collaboration" OR "citizen collaboration" OR "stakeholder collaboration" OR "representative collaboration" OR "relative collaboration" OR "family collaboration" OR "lay collaboration" OR "resident collaboration" OR "patient partnership" OR "client partnership" OR "consumer partnership" OR "user partnership" OR "carer partnership" OR "caregiver partnership" OR "public partnership" OR "citizen partnership" OR "stakeholder partnership" OR "representative partnership" OR "relative partnership" OR "family partnership" OR "lay partnership" OR "resident partnership" OR "patient empowerment" OR "client empowerment" OR "consumer empowerment" OR "user empowerment" OR "carer empowerment" OR "caregiver empowerment" OR "public empowerment" OR "citizen empowerment" OR "stakeholder empowerment" OR "representative empowerment" OR "relative empowerment" OR "family empowerment" OR "lay empowerment" OR "resident empowerment" OR "patient consultation" OR "client consultation" OR "consumer consultation" OR "user consultation" OR "carer consultation" OR "caregiver consultation" OR "public consultation" OR "citizen consultation" OR "stakeholder consultation" OR "representative consultation" OR "relative consultation" OR "family consultation" OR "lay consultation" OR "resident consultation" OR "Patient Activation" OR "Patient Activations" OR "co-research*" OR coresearch*) AND ("Residential Home" OR "Residential Facilities" OR "Residential Facility" OR "Nursing Home" OR "Nursing Homes" OR "Nursing Home" OR "Institutionalization" OR "institutional care" OR "Institutionalization" OR "Institutionalisation" OR "Long Term Care" OR "Long-Term Care" OR "nursing home patient" OR "Home resident" OR "Home residents" OR "Dementia" OR "dementia" OR dement* OR alzheimer* OR "Mental health care" OR "Mental healthcare" OR "Mental health care" OR "Mental Health Service" OR "Mental Health Services" OR "Mental Health Service" OR "Disabled Person" OR "Disabled" OR "Cognitive impair*" OR "Cognitive Defect") AND ("Research" OR "Medical research" OR "Medical research" OR "Biomedical research" OR "Care research" OR "Healthcare research" OR "Scientific research" OR "Research agenda" OR "Research agendas" OR "research priorities" OR "research priority" OR "Research programs" OR "Research program" OR "Research programmes" OR "Research programme" OR "Research methods" OR "Research method" OR "Research questions" OR "Research question" OR "Participatory research" OR "co research*" OR coresearch* OR "Behavioral Research" OR "Health Services Research" OR "Community-Based Participatory Research" OR "Comparative Effectiveness Research" OR "Global Burden of Disease" OR "Health Care Survey" OR "Health Care Surveys" OR "Health Impact Assessment" OR "Health Services Needs and Demand" OR "Needs Assessment" OR "Organizational Case Studies" OR "Organizational Case Study" OR "Patient Reported Outcome Measure" OR "Patient Reported Outcome Measures" OR "Human Experimentation" OR "Nursing Research" OR "Outcome Assessment" OR "Treatment Outcome" OR "Pharmaceutical Research" OR "Pharmacy Research" OR "Rehabilitation Research" OR "Community-Based Participatory Research" OR "Participatory Research" OR "Empirical Research" OR "Grounded Theory" OR "Qualitative Research" OR "Interdisciplinary Research" OR "Operations Research" OR "Public Health Systems Research" OR **"Methodology"** OR "Empirical Research" OR "Grounded Theory" OR "Qualitative Research" OR "Interdisciplinary Research" OR "Operations Research" OR "Peer Review" OR "Public Health Systems Research" OR "Research Design" OR "Research Report" OR "Research Subject" OR "Research Subjects" OR "Research Subject" OR "Participatory Research" **OR "research"**) AND (Reform* OR Develop* OR Change* OR Design* OR Redesign* OR "Conceptualization" OR "Conceptualisation" OR concept* OR Model* OR Method* OR "strategy" OR "strategies" OR strateg*))

1. **Sociological Abstracts**

NOFT(("patient participation" OR "patient participation" OR "client participation" OR "consumer participation" OR "user participation" OR "carer participation" OR "caregiver participation" OR "public participation" OR "citizen participation" OR "stakeholder participation" OR "representative participation" OR "relative participation" OR "family participation" OR "lay participation" OR "resident participation" OR "patient involvement" OR "client involvement" OR "consumer involvement" OR "user involvement" OR "carer involvement" OR "caregiver involvement" OR "public involvement" OR "citizen involvement" OR "stakeholder involvement" OR "representative involvement" OR "relative involvement" OR "family involvement" OR "lay involvement" OR "resident involvement" OR "patient engagement" OR "client engagement" OR "consumer engagement" OR "user engagement" OR "carer engagement" OR "caregiver engagement" OR "public engagement" OR "citizen engagement" OR "stakeholder engagement" OR "representative engagement" OR "relative engagement" OR "family engagement" OR "lay engagement" OR "resident engagement" OR "patient collaboration" OR "client collaboration" OR "consumer collaboration" OR "user collaboration" OR "carer collaboration" OR "caregiver collaboration" OR "public collaboration" OR "citizen collaboration" OR "stakeholder collaboration" OR "representative collaboration" OR "relative collaboration" OR "family collaboration" OR "lay collaboration" OR "resident collaboration" OR "patient partnership" OR "client partnership" OR "consumer partnership" OR "user partnership" OR "carer partnership" OR "caregiver partnership" OR "public partnership" OR "citizen partnership" OR "stakeholder partnership" OR "representative partnership" OR "relative partnership" OR "family partnership" OR "lay partnership" OR "resident partnership" OR "patient empowerment" OR "client empowerment" OR "consumer empowerment" OR "user empowerment" OR "carer empowerment" OR "caregiver empowerment" OR "public empowerment" OR "citizen empowerment" OR "stakeholder empowerment" OR "representative empowerment" OR "relative empowerment" OR "family empowerment" OR "lay empowerment" OR "resident empowerment" OR "patient consultation" OR "client consultation" OR "consumer consultation" OR "user consultation" OR "carer consultation" OR "caregiver consultation" OR "public consultation" OR "citizen consultation" OR "stakeholder consultation" OR "representative consultation" OR "relative consultation" OR "family consultation" OR "lay consultation" OR "resident consultation" OR "Patient Activation" OR "Patient Activations" OR "co-research*" OR coresearch*) AND ("Residential Home" OR "Residential Facilities" OR "Residential Facility" OR "Nursing Home" OR "Nursing Homes" OR "Nursing Home" OR "Institutionalization" OR "institutional care" OR "Institutionalization" OR "Institutionalisation" OR "Long Term Care" OR "Long-Term Care" OR "nursing home patient" OR "Home resident" OR "Home residents" OR "Dementia" OR "dementia" OR dement* OR alzheimer* OR "Mental health care" OR "Mental healthcare" OR "Mental health care" OR "Mental Health Service" OR "Mental Health Services" OR "Mental Health Service" OR "Disabled Person" OR "Disabled" OR "Cognitive impair*" OR "Cognitive Defect") AND ("Research" OR "Medical research" OR "Medical research" OR "Biomedical research" OR "Care research" OR "Healthcare research" OR "Scientific research" OR "Research agenda" OR "Research agendas" OR "research priorities" OR "research priority" OR "Research programs" OR "Research program" OR "Research programmes" OR "Research programme" OR "Research methods" OR "Research method" OR "Research questions" OR "Research question" OR "Participatory research" OR "co research*" OR coresearch* OR "Behavioral Research" OR "Health Services Research" OR "Community-Based Participatory Research" OR "Comparative Effectiveness Research" OR "Global Burden of Disease" OR "Health Care Survey" OR "Health Care Surveys" OR "Health Impact Assessment" OR "Health Services Needs and Demand" OR "Needs Assessment" OR "Organizational Case Studies" OR "Organizational Case Study" OR "Patient Reported Outcome Measure" OR "Patient Reported Outcome Measures" OR "Human Experimentation" OR "Nursing Research" OR "Outcome Assessment" OR "Treatment Outcome" OR "Pharmaceutical Research" OR "Pharmacy Research" OR "Rehabilitation Research" OR "Community-Based Participatory Research" OR "Participatory Research" OR "Empirical Research" OR "Grounded Theory" OR "Qualitative Research" OR "Interdisciplinary Research" OR "Operations Research" OR "Public Health Systems Research" OR **"Methodology"** OR "Empirical Research" OR "Grounded Theory" OR "Qualitative Research" OR "Interdisciplinary Research" OR "Operations Research" OR "Peer Review" OR "Public Health Systems Research" OR "Research Design" OR "Research Report" OR "Research Subject" OR "Research Subjects" OR "Research Subject" OR "Participatory Research" **OR "research"**) AND (Reform* OR Develop* OR Change* OR Design* OR Redesign* OR "Conceptualization" OR "Conceptualisation" OR concept* OR Model* OR Method* OR "strategy" OR "strategies" OR strateg*))

1. **Psychology and Behavioral Sciences Collection**

(TI(("patient participation" OR "patient participation" OR "client participation" OR "consumer participation" OR "user participation" OR "carer participation" OR "caregiver participation" OR "public participation" OR "citizen participation" OR "stakeholder participation" OR "representative participation" OR "relative participation" OR "family participation" OR "lay participation" OR "resident participation" OR "patient involvement" OR "client involvement" OR "consumer involvement" OR "user involvement" OR "carer involvement" OR "caregiver involvement" OR "public involvement" OR "citizen involvement" OR "stakeholder involvement" OR "representative involvement" OR "relative involvement" OR "family involvement" OR "lay involvement" OR "resident involvement" OR "patient engagement" OR "client engagement" OR "consumer engagement" OR "user engagement" OR "carer engagement" OR "caregiver engagement" OR "public engagement" OR "citizen engagement" OR "stakeholder engagement" OR "representative engagement" OR "relative engagement" OR "family engagement" OR "lay engagement" OR "resident engagement" OR "patient collaboration" OR "client collaboration" OR "consumer collaboration" OR "user collaboration" OR "carer collaboration" OR "caregiver collaboration" OR "public collaboration" OR "citizen collaboration" OR "stakeholder collaboration" OR "representative collaboration" OR "relative collaboration" OR "family collaboration" OR "lay collaboration" OR "resident collaboration" OR "patient partnership" OR "client partnership" OR "consumer partnership" OR "user partnership" OR "carer partnership" OR "caregiver partnership" OR "public partnership" OR "citizen partnership" OR "stakeholder partnership" OR "representative partnership" OR "relative partnership" OR "family partnership" OR "lay partnership" OR "resident partnership" OR "patient empowerment" OR "client empowerment" OR "consumer empowerment" OR "user empowerment" OR "carer empowerment" OR "caregiver empowerment" OR "public empowerment" OR "citizen empowerment" OR "stakeholder empowerment" OR "representative empowerment" OR "relative empowerment" OR "family empowerment" OR "lay empowerment" OR "resident empowerment" OR "patient consultation" OR "client consultation" OR "consumer consultation" OR "user consultation" OR "carer consultation" OR "caregiver consultation" OR "public consultation" OR "citizen consultation" OR "stakeholder consultation" OR "representative consultation" OR "relative consultation" OR "family consultation" OR "lay consultation" OR "resident consultation" OR "Patient Activation" OR "Patient Activations" OR "co-research*" OR coresearch*) AND ("Residential Home" OR "Residential Facilities" OR "Residential Facility" OR "Nursing Home" OR "Nursing Homes" OR "Nursing Home" OR "Institutionalization" OR "institutional care" OR "Institutionalization" OR "Institutionalisation" OR "Long Term Care" OR "Long-Term Care" OR "nursing home patient" OR "Home resident" OR "Home residents" OR exp "Dementia" OR "dementia" OR dement* OR alzheimer* OR exp "Mental health care" OR "Mental healthcare" OR "Mental health care" OR exp "Mental Health Service" OR "Mental Health Services" OR "Mental Health Service" OR exp "Disabled Person" OR "Disabled" OR "Cognitive impair*" OR exp "Cognitive Defect") AND (Reform* OR Develop* OR Change* OR Design* OR Redesign* OR "Conceptualization" OR "Conceptualisation" OR concept* OR Model* OR Method* OR "strategy" OR "strategies" OR strateg*)) AND TI("Research" OR "Medical research" OR "Medical research" OR "Biomedical research" OR "Care research" OR "Healthcare research" OR "Scientific research" OR "Research agenda" OR "Research agendas" OR "research priorities" OR "research priority" OR "Research programs" OR "Research program" OR "Research programmes" OR "Research programme" OR "Research methods" OR "Research method" OR "Research questions" OR "Research question" OR "Participatory research" OR "co research*" OR coresearch* OR "Behavioral Research" OR "Health Services Research" OR "Community-Based Participatory Research" OR "Comparative Effectiveness Research" OR "Global Burden of Disease" OR "Health Care Survey" OR "Health Care Surveys" OR "Health Impact Assessment" OR "Health Services Needs and Demand" OR "Needs Assessment" OR "Organizational Case Studies" OR "Organizational Case Study" OR "Patient Reported Outcome Measure" OR "Patient Reported Outcome Measures" OR "Human Experimentation" OR "Nursing Research" OR "Outcome Assessment" OR "Treatment Outcome" OR "Pharmaceutical Research" OR "Pharmacy Research" OR "Rehabilitation Research" OR "Community-Based Participatory Research" OR "Participatory Research" OR "Empirical Research" OR "Grounded Theory" OR "Qualitative Research" OR "Interdisciplinary Research" OR "Operations Research" OR "Public Health Systems Research" OR **"Methodology"** OR "Empirical Research" OR "Grounded Theory" OR "Qualitative Research" OR "Interdisciplinary Research" OR "Operations Research" OR "Peer Review, Research" OR "Public Health Systems Research" OR "Research Design" OR "Research Report" OR "Research Subject" OR "Research Subjects" OR "Research Subject" OR "Participatory Research" **OR "research"**)) OR (SU(("patient participation" OR "patient participation" OR "client participation" OR "consumer participation" OR "user participation" OR "carer participation" OR "caregiver participation" OR "public participation" OR "citizen participation" OR "stakeholder participation" OR "representative participation" OR "relative participation" OR "family participation" OR "lay participation" OR "resident participation" OR "patient involvement" OR "client involvement" OR "consumer involvement" OR "user involvement" OR "carer involvement" OR "caregiver involvement" OR "public involvement" OR "citizen involvement" OR "stakeholder involvement" OR "representative involvement" OR "relative involvement" OR "family involvement" OR "lay involvement" OR "resident involvement" OR "patient engagement" OR "client engagement" OR "consumer engagement" OR "user engagement" OR "carer engagement" OR "caregiver engagement" OR "public engagement" OR "citizen engagement" OR "stakeholder engagement" OR "representative engagement" OR "relative engagement" OR "family engagement" OR "lay engagement" OR "resident engagement" OR "patient collaboration" OR "client collaboration" OR "consumer collaboration" OR "user collaboration" OR "carer collaboration" OR "caregiver collaboration" OR "public collaboration" OR "citizen collaboration" OR "stakeholder collaboration" OR "representative collaboration" OR "relative collaboration" OR "family collaboration" OR "lay collaboration" OR "resident collaboration" OR "patient partnership" OR "client partnership" OR "consumer partnership" OR "user partnership" OR "carer partnership" OR "caregiver partnership" OR "public partnership" OR "citizen partnership" OR "stakeholder partnership" OR "representative partnership" OR "relative partnership" OR "family partnership" OR "lay partnership" OR "resident partnership" OR "patient empowerment" OR "client empowerment" OR "consumer empowerment" OR "user empowerment" OR "carer empowerment" OR "caregiver empowerment" OR "public empowerment" OR "citizen empowerment" OR "stakeholder empowerment" OR "representative empowerment" OR "relative empowerment" OR "family empowerment" OR "lay empowerment" OR "resident empowerment" OR "patient consultation" OR "client consultation" OR "consumer consultation" OR "user consultation" OR "carer consultation" OR "caregiver consultation" OR "public consultation" OR "citizen consultation" OR "stakeholder consultation" OR "representative consultation" OR "relative consultation" OR "family consultation" OR "lay consultation" OR "resident consultation" OR "Patient Activation" OR "Patient Activations" OR "co-research*" OR coresearch*) AND ("Residential Home" OR "Residential Facilities" OR "Residential Facility" OR "Nursing Home" OR "Nursing Homes" OR "Nursing Home" OR "Institutionalization" OR "institutional care" OR "Institutionalization" OR "Institutionalisation" OR "Long Term Care" OR "Long-Term Care" OR "nursing home patient" OR "Home resident" OR "Home residents" OR exp "Dementia" OR "dementia" OR dement* OR alzheimer* OR exp "Mental health care" OR "Mental healthcare" OR "Mental health care" OR exp "Mental Health Service" OR "Mental Health Services" OR "Mental Health Service" OR exp "Disabled Person" OR "Disabled" OR "Cognitive impair*" OR exp "Cognitive Defect") AND (Reform* OR Develop* OR Change* OR Design* OR Redesign* OR "Conceptualization" OR "Conceptualisation" OR concept* OR Model* OR Method* OR "strategy" OR "strategies" OR strateg*)) AND TI("Research" OR "Medical research" OR "Medical research" OR "Biomedical research" OR "Care research" OR "Healthcare research" OR "Scientific research" OR "Research agenda" OR "Research agendas" OR "research priorities" OR "research priority" OR "Research programs" OR "Research program" OR "Research programmes" OR "Research programme" OR "Research methods" OR "Research method" OR "Research questions" OR "Research question" OR "Participatory research" OR "co research*" OR coresearch* OR "Behavioral Research" OR "Health Services Research" OR "Community-Based Participatory Research" OR "Comparative Effectiveness Research" OR "Global Burden of Disease" OR "Health Care Survey" OR "Health Care Surveys" OR "Health Impact Assessment" OR "Health Services Needs and Demand" OR "Needs Assessment" OR "Organizational Case Studies" OR "Organizational Case Study" OR "Patient Reported Outcome Measure" OR "Patient Reported Outcome Measures" OR "Human Experimentation" OR "Nursing Research" OR "Outcome Assessment" OR "Treatment Outcome" OR "Pharmaceutical Research" OR "Pharmacy Research" OR "Rehabilitation Research" OR "Community-Based Participatory Research" OR "Participatory Research" OR "Empirical Research" OR "Grounded Theory" OR "Qualitative Research" OR "Interdisciplinary Research" OR "Operations Research" OR "Public Health Systems Research" OR **"Methodology"** OR "Empirical Research" OR "Grounded Theory" OR "Qualitative Research" OR "Interdisciplinary Research" OR "Operations Research" OR "Peer Review, Research" OR "Public Health Systems Research" OR "Research Design" OR "Research Report" OR "Research Subject" OR "Research Subjects" OR "Research Subject" OR "Participatory Research" **OR "research"**)) OR (MA(("patient participation" OR "patient participation" OR "client participation" OR "consumer participation" OR "user participation" OR "carer participation" OR "caregiver participation" OR "public participation" OR "citizen participation" OR "stakeholder participation" OR "representative participation" OR "relative participation" OR "family participation" OR "lay participation" OR "resident participation" OR "patient involvement" OR "client involvement" OR "consumer involvement" OR "user involvement" OR "carer involvement" OR "caregiver involvement" OR "public involvement" OR "citizen involvement" OR "stakeholder involvement" OR "representative involvement" OR "relative involvement" OR "family involvement" OR "lay involvement" OR "resident involvement" OR "patient engagement" OR "client engagement" OR "consumer engagement" OR "user engagement" OR "carer engagement" OR "caregiver engagement" OR "public engagement" OR "citizen engagement" OR "stakeholder engagement" OR "representative engagement" OR "relative engagement" OR "family engagement" OR "lay engagement" OR "resident engagement" OR "patient collaboration" OR "client collaboration" OR "consumer collaboration" OR "user collaboration" OR "carer collaboration" OR "caregiver collaboration" OR "public collaboration" OR "citizen collaboration" OR "stakeholder collaboration" OR "representative collaboration" OR "relative collaboration" OR "family collaboration" OR "lay collaboration" OR "resident collaboration" OR "patient partnership" OR "client partnership" OR "consumer partnership" OR "user partnership" OR "carer partnership" OR "caregiver partnership" OR "public partnership" OR "citizen partnership" OR "stakeholder partnership" OR "representative partnership" OR "relative partnership" OR "family partnership" OR "lay partnership" OR "resident partnership" OR "patient empowerment" OR "client empowerment" OR "consumer empowerment" OR "user empowerment" OR "carer empowerment" OR "caregiver empowerment" OR "public empowerment" OR "citizen empowerment" OR "stakeholder empowerment" OR "representative empowerment" OR "relative empowerment" OR "family empowerment" OR "lay empowerment" OR "resident empowerment" OR "patient consultation" OR "client consultation" OR "consumer consultation" OR "user consultation" OR "carer consultation" OR "caregiver consultation" OR "public consultation" OR "citizen consultation" OR "stakeholder consultation" OR "representative consultation" OR "relative consultation" OR "family consultation" OR "lay consultation" OR "resident consultation" OR "Patient Activation" OR "Patient Activations" OR "co-research*" OR coresearch*) AND ("Residential Home" OR "Residential Facilities" OR "Residential Facility" OR "Nursing Home" OR "Nursing Homes" OR "Nursing Home" OR "Institutionalization" OR "institutional care" OR "Institutionalization" OR "Institutionalisation" OR "Long Term Care" OR "Long-Term Care" OR "nursing home patient" OR "Home resident" OR "Home residents" OR exp "Dementia" OR "dementia" OR dement* OR alzheimer* OR exp "Mental health care" OR "Mental healthcare" OR "Mental health care" OR exp "Mental Health Service" OR "Mental Health Services" OR "Mental Health Service" OR exp "Disabled Person" OR "Disabled" OR "Cognitive impair*" OR exp "Cognitive Defect") AND (Reform* OR Develop* OR Change* OR Design* OR Redesign* OR "Conceptualization" OR "Conceptualisation" OR concept* OR Model* OR Method* OR "strategy" OR "strategies" OR strateg*)) AND TI("Research" OR "Medical research" OR "Medical research" OR "Biomedical research" OR "Care research" OR "Healthcare research" OR "Scientific research" OR "Research agenda" OR "Research agendas" OR "research priorities" OR "research priority" OR "Research programs" OR "Research program" OR "Research programmes" OR "Research programme" OR "Research methods" OR "Research method" OR "Research questions" OR "Research question" OR "Participatory research" OR "co research*" OR coresearch* OR "Behavioral Research" OR "Health Services Research" OR "Community-Based Participatory Research" OR "Comparative Effectiveness Research" OR "Global Burden of Disease" OR "Health Care Survey" OR "Health Care Surveys" OR "Health Impact Assessment" OR "Health Services Needs and Demand" OR "Needs Assessment" OR "Organizational Case Studies" OR "Organizational Case Study" OR "Patient Reported Outcome Measure" OR "Patient Reported Outcome Measures" OR "Human Experimentation" OR "Nursing Research" OR "Outcome Assessment" OR "Treatment Outcome" OR "Pharmaceutical Research" OR "Pharmacy Research" OR "Rehabilitation Research" OR "Community-Based Participatory Research" OR "Participatory Research" OR "Empirical Research" OR "Grounded Theory" OR "Qualitative Research" OR "Interdisciplinary Research" OR "Operations Research" OR "Public Health Systems Research" OR **"Methodology"** OR "Empirical Research" OR "Grounded Theory" OR "Qualitative Research" OR "Interdisciplinary Research" OR "Operations Research" OR "Peer Review, Research" OR "Public Health Systems Research" OR "Research Design" OR "Research Report" OR "Research Subject" OR "Research Subjects" OR "Research Subject" OR "Participatory Research" **OR "research"**)) OR (AB(("patient participation" OR "patient participation" OR "client participation" OR "consumer participation" OR "user participation" OR "carer participation" OR "caregiver participation" OR "public participation" OR "citizen participation" OR "stakeholder participation" OR "representative participation" OR "relative participation" OR "family participation" OR "lay participation" OR "resident participation" OR "patient involvement" OR "client involvement" OR "consumer involvement" OR "user involvement" OR "carer involvement" OR "caregiver involvement" OR "public involvement" OR "citizen involvement" OR "stakeholder involvement" OR "representative involvement" OR "relative involvement" OR "family involvement" OR "lay involvement" OR "resident involvement" OR "patient engagement" OR "client engagement" OR "consumer engagement" OR "user engagement" OR "carer engagement" OR "caregiver engagement" OR "public engagement" OR "citizen engagement" OR "stakeholder engagement" OR "representative engagement" OR "relative engagement" OR "family engagement" OR "lay engagement" OR "resident engagement" OR "patient collaboration" OR "client collaboration" OR "consumer collaboration" OR "user collaboration" OR "carer collaboration" OR "caregiver collaboration" OR "public collaboration" OR "citizen collaboration" OR "stakeholder collaboration" OR "representative collaboration" OR "relative collaboration" OR "family collaboration" OR "lay collaboration" OR "resident collaboration" OR "patient partnership" OR "client partnership" OR "consumer partnership" OR "user partnership" OR "carer partnership" OR "caregiver partnership" OR "public partnership" OR "citizen partnership" OR "stakeholder partnership" OR "representative partnership" OR "relative partnership" OR "family partnership" OR "lay partnership" OR "resident partnership" OR "patient empowerment" OR "client empowerment" OR "consumer empowerment" OR "user empowerment" OR "carer empowerment" OR "caregiver empowerment" OR "public empowerment" OR "citizen empowerment" OR "stakeholder empowerment" OR "representative empowerment" OR "relative empowerment" OR "family empowerment" OR "lay empowerment" OR "resident empowerment" OR "patient consultation" OR "client consultation" OR "consumer consultation" OR "user consultation" OR "carer consultation" OR "caregiver consultation" OR "public consultation" OR "citizen consultation" OR "stakeholder consultation" OR "representative consultation" OR "relative consultation" OR "family consultation" OR "lay consultation" OR "resident consultation" OR "Patient Activation" OR "Patient Activations" OR "co-research*" OR coresearch*) AND ("Residential Home" OR "Residential Facilities" OR "Residential Facility" OR "Nursing Home" OR "Nursing Homes" OR "Nursing Home" OR "Institutionalization" OR "institutional care" OR "Institutionalization" OR "Institutionalisation" OR "Long Term Care" OR "Long-Term Care" OR "nursing home patient" OR "Home resident" OR "Home residents" OR exp "Dementia" OR "dementia" OR dement* OR alzheimer* OR exp "Mental health care" OR "Mental healthcare" OR "Mental health care" OR exp "Mental Health Service" OR "Mental Health Services" OR "Mental Health Service" OR exp "Disabled Person" OR "Disabled" OR "Cognitive impair*" OR exp "Cognitive Defect") AND (Reform* OR Develop* OR Change* OR Design* OR Redesign* OR "Conceptualization" OR "Conceptualisation" OR concept* OR Model* OR Method* OR "strategy" OR "strategies" OR strateg*)) AND TI("Research" OR "Medical research" OR "Medical research" OR "Biomedical research" OR "Care research" OR "Healthcare research" OR "Scientific research" OR "Research agenda" OR "Research agendas" OR "research priorities" OR "research priority" OR "Research programs" OR "Research program" OR "Research programmes" OR "Research programme" OR "Research methods" OR "Research method" OR "Research questions" OR "Research question" OR "Participatory research" OR "co research*" OR coresearch* OR "Behavioral Research" OR "Health Services Research" OR "Community-Based Participatory Research" OR "Comparative Effectiveness Research" OR "Global Burden of Disease" OR "Health Care Survey" OR "Health Care Surveys" OR "Health Impact Assessment" OR "Health Services Needs and Demand" OR "Needs Assessment" OR "Organizational Case Studies" OR "Organizational Case Study" OR "Patient Reported Outcome Measure" OR "Patient Reported Outcome Measures" OR "Human Experimentation" OR "Nursing Research" OR "Outcome Assessment" OR "Treatment Outcome" OR "Pharmaceutical Research" OR "Pharmacy Research" OR "Rehabilitation Research" OR "Community-Based Participatory Research" OR "Participatory Research" OR "Empirical Research" OR "Grounded Theory" OR "Qualitative Research" OR "Interdisciplinary Research" OR "Operations Research" OR "Public Health Systems Research" OR **"Methodology"** OR "Empirical Research" OR "Grounded Theory" OR "Qualitative Research" OR "Interdisciplinary Research" OR "Operations Research" OR "Peer Review, Research" OR "Public Health Systems Research" OR "Research Design" OR "Research Report" OR "Research Subject" OR "Research Subjects" OR "Research Subject" OR "Participatory Research" **OR "research"**))
